# Relationship between QTL for grain shape, grain weight, test weight, milling yield, and plant height in the spring wheat cross RL4452/‘AC Domain’

**DOI:** 10.1371/journal.pone.0190681

**Published:** 2018-01-22

**Authors:** Adrian L. Cabral, Mark C. Jordan, Gary Larson, Daryl J. Somers, D. Gavin Humphreys, Curt A. McCartney

**Affiliations:** 1 Agriculture and Agri-Food Canada, Morden Research and Development Centre, Morden, Manitoba, Canada; 2 Agriculture and Agri-Food Canada, Lethbridge Research and Development Centre, Lethbridge, Alberta, Canada; 3 Vineland Research and Innovation Centre, Vineland Station, Ontario, Canada; 4 Agriculture and Agri-Food Canada, Ottawa Research and Development Centre, Ottawa, Ontario, Canada; Institute of Genetics and Developmental Biology Chinese Academy of Sciences, CHINA

## Abstract

Kernel morphology characteristics of wheat are complex and quantitatively inherited. A doubled haploid (DH) population of the cross RL4452/‘AC Domain’ was used to study the genetic basis of seed shape. Quantitative trait loci (QTL) analyses were conducted on a total of 18 traits: 14 grain shape traits, flour yield (Fyd), and three agronomic traits (Plant height [Plht], 1000 Grain weight [Gwt], Test weight [Twt]), using data from trial locations at Glenlea, Brandon, and Morden in Manitoba, Canada, between 1999 and 2004. Kernel shape was studied through digital image analysis with an Acurum® grain analyzer. Plht, Gwt, Twt, Fyd, and grain shape QTL were correlated with each other and QTL analysis revealed that QTL for these traits often mapped to the same genetic locations. The most significant QTL for the grain shape traits were located on chromosomes 4B and 4D, each accounting for up to 24.4% and 53.3% of the total phenotypic variation, respectively. In addition, the most significant QTL for Plht, Gwt, and Twt were all detected on chromosome 4D at the *Rht-D1* locus. *Rht-D1b* decreased Plht, Gwt, Twt, and kernel width relative to the *Rht-D1a* allele. A narrow genetic interval on chromosome 4B contained significant QTL for grain shape, Gwt, and Plht. The ‘AC Domain’ allele reduced Plht, Gwt, kernel length and width traits, but had no detectable effect on Twt. The data indicated that this variation was inconsistent with segregation at *Rht-B1*. Numerous QTL were identified that control these traits in this population.

## Introduction

Wheat (*Tritcium aestivum* L.) is an allohexaploid species (2n = 6x = 42) comprised of A, B, and D sub-genomes totalling ~17 Gbp. Along with other important cereal crops, it has been subject to artificial selection for increased grain size since the early stages of its cultivation [1]. Size and shape of wheat kernels affect kernel weight and test weight [2], besides also influencing milling yields and grain protein content [3]. Both milling yields and grain protein content traits are distinct and independent of each other [4].

Grain or kernel size of wheat is most often described by grain-length and grain-width parameters. Although QTL for grain size and/or grain shape have been identified on almost all wheat chromosomes [2, 4–10], only a few of the underlying genes influencing grain size or shape have been cloned. The grain size locus *TaGS-D1* on chromosome 7DS, associated with grain length and grain weight is an ortholog of the *OsGs3* gene located on chromosome 3 of rice [11, 12]. A second locus *TaGw2*, controlling grain width and grain weight is located on chromosome 6A of wheat [13], and is an ortholog of the *GW2* locus controlling grain weight on chromosome 2 of rice [14]. Besides the two grain weight loci, another locus for grain weight *TaCKX6-D1* on chromosome 3D of wheat was cloned [15], and is an ortholog of *OsCKX2* located on chromosome 1 of rice [16]. On rice chromosome 5, grain size and grain width loci *GS5* [17] and *GW5* [18, 19] have been cloned and functionally characterized.

Semi-dwarf wheat varieties were first released in the US in 1961 (‘Gaines’), and later in Mexico in 1962 (‘Pitic 62’, ‘Penjamo 62’), and in 1964 (‘Sonora 64’, ‘Lerma Rojo 64’, ‘Super X’, ‘Siete Cerros’). All the above varieties contained either one or two of the dwarfing/reduced height genes (*Rht1*, *Rht2*) derived from the Japanese winter wheat variety Norin 10 [20, 21]. These two gibberellic acid-insensitive genes *Rht1* (*Rht-B1*) and *Rht2* (*Rht-D1*) [22, 23] located on chromosomes 4B and 4D, respectively [24, 25], have been studied extensively. In addition, their wild type and mutant alleles have also been cloned [26]. The same RL4452/‘AC Domain’ DH population used in this study identified Plht QTL near the expected locations of *Rht-B1* and *Rht-D1* on chromosomes 4B and 4D, respectively [27]. Several other studies have investigated relationships between *Rht* genes and yield/yield components: [28–33]. Pleiotrophic effects of *Rht* genotype on coleoptile length, early vigour, and dry matter partitioning [34–36] and on grain shape (*Rht8*) [37] have also been reported.

Our objectives were to: a) identify significant grain morphology and agronomic trait QTL (Plht, Gwt, Twt, Fyd), and b) determine their interrelationships.

## Materials and methods

### Plant material

A total of 193 DH progeny genotypes derived from a cross between Canadian spring wheats RL4452/‘AC Domain’ were used in the construction of a genetic linkage map. ‘AC Domain’ was a widely grown cultivar, which was registered in the Canada Western Red Spring (CWRS) marketing class in 1992 [38]. ‘AC Domain’ has the pedigree BW83/ND585 (alternatively, ND499/RL4137//ND585). It is a prominent parent in western Canadian spring wheat breeding because of its excellent pre-harvest sprouting resistance [39]. RL4452 (pedigree: ‘Glenlea’*6/‘Kitt’) is an unregistered backcross derivative of the wheat cultivar ‘Glenlea’ with the dwarfing gene *Rht-D1b* introgressed from Kitt. ‘Glenlea’ [40] was the quality standard for the Canada Western Extra Strong (CWES) marketing class. ‘Kitt’ is a semi-dwarf hard red spring wheat released by the University of Minnesota in 1975. QTL mapping was carried out using 183 DH progeny genotypes for which trait data was available.

### Grain shape traits

An Acurum® grain analyzer was used to evaluate 14 grain shape traits on the RL4452/‘AC Domain’ DH population (Table 1). Details regarding the Acurum® grain analyzer were outlined in US Patent 7,218,775 B2, “Method and apparatus for identifying and quantifying characteristics of seeds and other small objects” [41]. The Acurum system consists of image capture of the sample (i.e. grain) and neural network analysis. Both average and standard deviation values for grain shape traits were calculated. A plot-wise analysis of grain traits with the Acurum® grain analyzer permitted calculation of average values (for all of the grains per plot) that were used for detecting QTL. Standard deviation values for grain shape were included to study variability in grain size and/or shape within grain samples (i.e. possibly from tillers or fertile tertiary florets).

**Table 1:** Grain shape traits measured on wheat grain samples with the Acurum® grain analyzer.

Abbreviation	Trait	Description
AMaL	Axis Major Length	Length of major axis
AMiL	Axis Minor Length	Length of minor axis
Area	Area	Grain area
ArPe	Area/Perimeter	Ratio of grain area/perimeter
Asym	Asymmetry	Grain shape symmetry
DMax	Diameter Max	Maximum diameter of the grain
DMen	Mean Diameter	Mean diameter of the grain
DMin	Diameter Min	Minimum diameter of the grain
Per	Perimeter	Perimeter of the grain
Rect	Rectangularity	Measurement of how closely a grain resembles a rectangle; a ratio of an object to its minimum bounding rectangle
Rndn	Roundness	How close the grain resembles a circle
Sphr	Sphericity	Measures the roundness of an object; a sphere will have a value of 1, while other shapes less than 1
SzLn	Size-Length	Maximum length of the grain
SzWd	Size-Width	Maximum width of the grain

### Plant height, grain weight, test weight, and flour yield (Plht, Gwt, Twt and Fyd)

Data on Plht was obtained from field trials at Glenlea (1998, 1999, and 2000) and Morden (1998, 1999, and 2000) in Manitoba, Canada. Gwt and Twt measurement were carried out using grain harvested from trials at Glenlea (1999 and 2000) and Morden (1999 and 2000) as described in McCartney et al. (2005). LS means for Gwt and Twt were used for QTL detection. Similarly, data for Fyd was collected and previously reported in McCartney et al. (2006). Grain samples were milled into straight-grade flour with a Buhler laboratory automatic-pneumatic mill (Model 202, Buhler AG, Uzwil, Switzerland) after tempering to 16.5% moisture. Flour yield was calculated based on total recovered products.

### Statistical analyses of trait data

Analysis of variance (ANOVA) was conducted with the GLM procedure of SAS® 9.3 (SAS Institute Inc., Cary, North Carolina, USA) with environments, replicates, and genotypes as random effects. Heritability was calculated on an entry mean and per plot basis with the ANOVA mean squares and the expectations of mean squares. Genotype line means were calculated for the agronomic traits with the LSMEANS statement of the MIXED procedure, which calculates least-square means. In this case, genotypes were considered fixed effects, while with environments and replicates were random effects. An overall mean dataset was generated for all traits by averaging trait data over all replicates. Correlation analysis was used to investigate potential genetic relationships between the traits. Pearson’s correlation coefficients were estimated between the agronomic, milling, and seed shape traits with procedure CORR of SAS® using the DH line means from the overall mean dataset.

### Linkage mapping and QTL analyses

Linkage and QTL mapping procedures for this experiment have been previously detailed [39]. In brief, an initial of 12,351 polymorphic markers (SSR, SNP, Diversity Arrays Technology [DArT], and ESTs) of an Illumina wheat 90K Infinium Custom beadchip [42] were screened on 193 DH progeny of the RL4452/‘AC Domain’ population. A total of 12,202 informative markers were used for linkage mapping with MapDisto® [43]. Linkage groups were identified using a minimum LOD score of 4, and a maximum recombination fraction of 0.25. Recombination fractions were converted into centiMorgan (cM) map distances using the Kosambi mapping function. The RL4452/‘AC Domain’ linkage map is reported in S1 Table. More than one linkage group was obtained for chromosomes 1B, 2B, 3D, 5A, 5D, 6D, 7B, and 7D. For instance, there were two linkage groups for chromosome 1B. Linkage group 1B.1 consisted of the short arm and most of the long arm, and linkage group 1B.2 consisted of the distal end of the long arm.

The most informative marker per linkage bin was utilized for QTL analyses (i.e. 1,055 markers were retained). QTL IciMapping software version 4.1.0.0 was used to test for additive effect and epistatic QTL from multi-year trial datasets using inclusive composite interval mapping (ICIM) [44]. Additive effect QTL were detected by ICIM (QIC module) with a walk speed of 0.1 cM. LOD thresholds were based on 1,000 permutations. The confidence interval was determined by one LOD drop-off, which approximates a 96.8% confidence interval [45]. Epistatic QTL were identified via a two-dimensional scan for mapping digenic epistasis using ICIM-epistasis (QICE module) with default LOD scores of 5.0, coupled with walk speeds of 2 cM. QTL were deemed significant if For agronomic and milling traits, QTL were reported when the peak LOD score exceeded the significance threshold determined by the permutation analyses in two or more environments. For seed shape traits, QTL were reported when the peak LOD score exceeded the significance threshold determined by permutation in a minimum of three combinations of shape traits by environment (Glenlea 2000, Brandon 2004, or meaned over both years). The phenotypic variation explained due to respective QTL was derived from marker-trait regression (r^2^) values.

### Physical locations of SNP markers

The physical locations of SNP markers were obtained with a BLASTN search against the IWGSC Chinese Spring RefSeq v1.0 database (https://urgi.versailles.inra.fr/blast_iwgsc/blast.php). The best BLAST hit for a SNP marker was reported for the chromosome to which it mapped in the RL4452/‘AC Domain’ DH population. The BLAST hits are reported in S1 Table.

## Results

Descriptive statistics of the seed shape, agronomic, and milling traits analyzed in the RL4452/ ‘AC Domain’ DH population are presented in S2 Table. Seed shape traits had high heritability estimates. The traits based upon mean seed shape parameters had heritabilities on a per plot basis ranging from 0.82 to 0.88, which was comparable to per plot heritability for test weight but exceeded the per plot heritability of plant height, grain weight, and flour yield.

Correlation analysis revealed the interrelationship between the traits assessed in the RL4452/‘AC Domain’ DH population (S3 Table). All seed shape traits were correlated with Gwt, with some being strongly correlated with Gwt (r > 0.9). Gwt was strongly positively correlated with kernel width (AmiL_M, DMin_M, SzWd_M), which also included mean seed diameter (DMen_M). There were also strong positive correlations between Gwt and seed area (Area_M), and Gwt and seed area-perimeter ratio (ArPe_M). Gwt was also highly correlated with seed length (AmaL_M, DMax_M, SzLn_M), but less so relative to kernel width. Plht and Twt were positively correlated with kernel width, but were not significantly correlated with kernel length. Flour yield (Fyd) was not strongly correlated with any trait, although statistically significant correlations were identified. Fyd was most strongly correlated with Sphericity (Sphr; r = -0.328). Rectangularity (Rect) had a strong positive correlation with Sphericity (Sphr; r = 0.986), and a strong negative correlation with Roundness (Rndn; r = -0.993). Kernel Area (Area_M) was correlated with all traits, except Rndn and Sphr. Kernel Perimeter (Per_M) was correlated with all traits, except Twt, Rect, and Rndn. Seed area-perimeter ratio (ArPe_M) was correlated with all traits to a certain degree.

Additive effect QTL for agronomic traits (Plht, Gwt, Twt) and flour yield (Fyd) are reported in Table 2, while additive effect QTL for kernel shape traits are outlined in Table 3. Agronomic trait QTL were detected on chromosomes 4B and 4D (Plht), 2B, 3B, 3D, 4A, 4B, 4D, 6B (Gwt), 1D, 2A, 2B, 2D, 3B, 3D, 4D, 7A (Twt), and 1B, 3B, 3D, 4B, 7D (Fyd). The most significant agronomic trait QTL *QPlht*.*crc-4D*, *QGwt*.*crc-4D*, and *QTwt*.*crc-4D* were all detected on chromosome 4D. Another notable QTL region was detected on chromosome 4B at 54 cM. This region affected Plht, Gwt, Fyd, and numerous grain shape parameters. The most significant QTL for Fyd were *QFyd*.*crc-3B* and *QFyd*.*crc-7D* on chromosomes 3B and 7D, respectively. QTL for kernel morphology traits were identified on 16 of the 21 wheat chromosomes. The most significant QTL (explaining the highest % phenotypic variation) for grain shape traits were identified on chromosomes 4B and 4D near the corresponding plant height (Plht) QTL.

**Table 2:** Inclusive Composite Interval Mapping (QIC) of Plant height (Plht), Grain weight (Gwt), Test weight (Twt), and Flour yield (Fyd) QTL identified in the RL4452/‘AC Domain’ DH population grown in replicated multi-year trials.

QTL	Trait name^a^	Chr	Peak (cM)	CI (cM)^b^	LOD	r^2^ (%)	Add^c^	Left marker	Right marker	LOD threshold (α_0.05_)
**Plant Height (Plht)**										
QPlht.crc-4B	Ht_MOR99	4B	52.9	52.4–53.5	6.7	9.9	-2.9	Tdurum_contig5562_441	TA003708-0300	3.11
QPlht.crc-4B	Ht_GLE99	4B	54.2	52.9–54.2	6.0	6.2	-2.5	BS00066282_51	wmc657	3.18
QPlht.crc-4B	Ht_GLE98	4B	54.3	54.2–55.5	13.1	15.6	-3.6	wmc657	Excalibur_c21727_851	3.06
QPlht.crc-4B	Ht_avg	4B	54.8	54.2–55.8	15.3	14.4	-2.9	wmc657	Excalibur_c21727_851	3.19
QPlht.crc-4B	Ht_BRA98	4B	54.8	54.2–55.7	7.5	10.4	-2.9	wmc657	Excalibur_c21727_851	3.08
QPlht.crc-4B	Ht_BRA00	4B	54.8	54.2–55.7	11.9	13.9	-2.7	wmc657	Excalibur_c21727_851	3.07
QPlht.crc-4B	Ht_GLE00	4B	54.8	54.2–55.9	7.6	8.8	-2.3	wmc657	Excalibur_c21727_851	3.10
QPlht.crc-4B	Ht_MOR00	4B	54.8	54.2–55.9	8.5	11.1	-2.4	wmc657	Excalibur_c21727_851	3.16
QPlht.crc-4B	Ht_MOR98	4B	55.9	54.8–58.6	5.5	13.3	-4.1	Excalibur_c21727_851	RAC875_rep_c98992_464	3.20
QPlht.crc-4D	Ht_MOR99	4D	32.8	28.9–36.2	24.0	51.0	6.6	wmc617c	wMAS000002	3.11
QPlht.crc-4D	Ht_MOR98	4D	33.4	25.9–37.2	10.0	26.8	5.8	wmc617c	wMAS000002	3.20
QPlht.crc-4D	Ht_avg	4D	34.2	32.1–36.1	38.3	58.6	5.8	wmc617c	wMAS000002	3.19
QPlht.crc-4D	Ht_BRA98	4D	34.2	31.4–36.4	25.4	50.7	6.3	wmc617c	wMAS000002	3.08
QPlht.crc-4D	Ht_GLE99	4D	34.2	32.0–37.1	32.8	57.9	7.6	wmc617c	wMAS000002	3.18
QPlht.crc-4D	Ht_BRA00	4D	34.2	32.5–35.9	29.5	44.5	4.9	wmc617c	wMAS000002	3.07
QPlht.crc-4D	Ht_GLE00	4D	34.2	32.4–35.7	30.4	49.7	5.4	wmc617c	wMAS000002	3.10
QPlht.crc-4D	Ht_MOR00	4D	34.3	32.7–37.2	26.5	45.5	4.9	wMAS000002	wmc48b	3.16
QPlht.crc-4D	Ht_GLE98	4D	35.2	32.7–39.0	28.2	46.8	6.3	wMAS000002	wmc48b	3.06
**Grain Weight (Gwt)**										
QGwt.crc-2B.1	Gwt_GLE00	2B.1	55.4	54.8–56.5	4.2	4.8	-0.7	RAC875_c31358_214	Tdurum_contig42153_4272	3.05
QGwt.crc-2B.1	Gwt_BRA04	2B.1	65.3	64.4–66.0	8.5	5.6	-1.1	wsnp_Ex_c21092_30220702	Excalibur_c6502_397	3.14
QGwt.crc-2B.1	Gwt_avg	2B.1	67.9	67.2–69.5	4.6	6.7	-0.9	RFL_Contig914_2723	BS00030497_51	3.28
QGwt.crc-2B.2	Gwt_MOR00	2B.1	80	78.6–81.0	10.8	9.6	-0.9	RFL_Contig1953_583	wsnp_CAP11_c114_140053	3.15
QGwt.crc-2B.2	Gwt_MOR99	2B.1	86.8	84.0–87.8	7.2	7.9	-1.2	Tdurum_contig26542_281	wsnp_Ex_rep_c105551_89940311	3.09
QGwt.crc-2B.2	Gwt_GLE00	2B.1	94.9	92.9–96.0	3.2	3.6	-0.6	wmc500b	wsnp_Ex_c9729_16071358	3.05
QGwt.crc-3B	Gwt_GLE99	3B	0	0–0.6	8.3	8.2	-1.3	Tdurum_contig50954_1393	Kukri_c15654_309	3.11
QGwt.crc-3B	Gwt_GLE00	3B	0	0–0.6	6.3	7.5	-0.9	Tdurum_contig50954_1393	Kukri_c15654_309	3.05
QGwt.crc-3D	Gwt_MOR99	3D.2	4.9	3.6–5.3	5.8	6.2	-1.1	tplb0029j24_2118	wsnp_Ex_rep_c101732_87042471	3.09
QGwt.crc-3D	Gwt_GLE99	3D.2	5.3	4.1–5.9	4.5	4.2	-0.9	wsnp_Ex_rep_c101732_87042471	Kukri_c8913_385	3.11
QGwt.crc-3D	Gwt_GLE00	3D.2	20.9	12.4–33.8	4.2	6.2	-0.8	BobWhite_c23305_1192	wmc552	3.05
QGwt.crc-4A	Gwt_avg	4A	90.1	89.5–92.8	3.4	4.9	0.8	Excalibur_c4325_1150	RAC875_c59673_500	3.28
QGwt.crc-4A	Gwt_MOR99	4A	90.1	89.5–92.7	6.6	7.2	1.2	Excalibur_c4325_1150	RAC875_c59673_500	3.09
QGwt.crc-4A	Gwt_MOR00	4A	90.1	89.5–92.4	6.4	5.3	0.6	Excalibur_c4325_1150	RAC875_c59673_500	3.15
QGwt.crc-4A	Gwt_BRA04	4A	90.1	89.5–92.8	6.3	4.0	1.0	Excalibur_c4325_1150	RAC875_c59673_500	3.14
QGwt.crc-4B	Gwt_MOR00	4B	51.4	51.3–52.4	15.8	15.1	-1.1	BS00105308_51	Tdurum_contig29989_132	3.15
QGwt.crc-4B	Gwt_MOR99	4B	51.9	51.3–52.4	12.2	14.9	-1.7	Tdurum_contig29989_132	Tdurum_contig5562_441	3.09
QGwt.crc-4B	Gwt_avg	4B	52.4	51.8–52.9	8.9	14.0	-1.3	Tdurum_contig29989_132	Tdurum_contig5562_441	3.28
QGwt.crc-4B	Gwt_GLE00	4B	52.4	51.8–52.9	6.3	7.4	-0.9	Tdurum_contig29989_132	Tdurum_contig5562_441	3.05
QGwt.crc-4B	Gwt_BRA04	4B	52.4	51.9–52.9	21.8	17.4	-2.0	Tdurum_contig29989_132	Tdurum_contig5562_441	3.14
QGwt.crc-4B	Gwt_GLE99	4B	52.5	52.4–53.4	7.8	7.5	-1.2	Tdurum_contig5562_441	TA003708-0300	3.11
QGwt.crc-4D	Gwt_avg	4D	31.8	27.3–34.3	16.5	31.1	2.0	wmc617c	wMAS000002	3.28
QGwt.crc-4D	Gwt_MOR99	4D	33.3	28.2–37.4	19.5	27.9	2.3	wmc617c	wMAS000002	3.09
QGwt.crc-4D	Gwt_GLE99	4D	34.2	30.2–36.5	29.4	45.0	3.0	wmc617c	wMAS000002	3.11
QGwt.crc-4D	Gwt_GLE00	4D	34.2	31.0–38.3	19.7	28.4	1.7	wmc617c	wMAS000002	3.05
QGwt.crc-4D	Gwt_MOR00	4D	34.2	31.3–37.1	25.9	28.8	1.5	wmc617c	wMAS000002	3.15
QGwt.crc-4D	Gwt_BRA04	4D	34.2	31.4–35.9	34.6	33.4	2.8	wmc617c	wMAS000002	3.14
QGwt.crc-6B	Gwt_BRA04	6B	139.4	135.7–142.1	5.1	3.4	0.9	RAC875_c6813_168	BS00049082_51	3.14
QGwt.crc-6B	Gwt_GLE99	6B	159.1	158.6–159.1	3.2	2.9	0.8	Tdurum_contig68258_1773	Kukri_c30924_203	3.11
**Test weight (Twt)**										
QTwt.crc-1D	Twt_MOR99	1D	96.9	96.1–97.5	9.8	11.3	-0.8	IAAV724	gpw0360	3.06
QTwt.crc-1D	Twt_MOR00	1D	111	97.7–124.9	4.7	6.0	-0.6	gpw0360	BS00022188_51	3.03
QTwt.crc-1D	Twt_BRA04	1D	112.3	103.0–123.2	6.2	5.9	-0.8	gpw0360	BS00022188_51	3.15
QTwt.crc-1D	Twt_GLE00	1D	117.3	104.1–117.2	6.2	7.0	-0.7	gpw0360	BS00022188_51	3.02
QTwt.crc-2A	Twt_GLE00	2A	89.6	88.9–92.2	3.4	3.3	-0.5	Kukri_rep_c68300_216	RAC875_c9523_328	3.02
QTwt.crc-2A	Twt_MOR00	2A	89.6	88.9–92.2	3.2	3.3	-0.5	Kukri_rep_c68300_216	RAC875_c9523_328	3.03
QTwt.crc-2B	Twt_MOR00	2B.1	27.6	26.4–36.0	5.5	6.0	-0.6	gpw5229	Excalibur_c40567_1893	3.03
QTwt.crc-2B	Twt_BRA04	2B.1	33.4	27.9–36.8	14.2	12.2	-1.1	gpw5229	Excalibur_c40567_1893	3.15
QTwt.crc-2B	Twt_GLE00	2B.1	39	36.7–42.3	3.0	2.9	-0.4	GENE-1999_98	wPt-8404	3.02
QTwt.crc-2D	Twt_BRA04	2D	88.9	87.3–92.6	3.4	2.5	0.5	gpw5256	Kukri_c92104_87	3.15
QTwt.crc-2D	Twt_MOR99	2D	102.7	100.9–104.3	3.1	3.1	0.4	wsnp_Ku_c498_1036380	Kukri_c52608_142	3.06
QTwt.crc-3B	Twt_BRA04	3B	60.1	58.8–61.2	11.5	9.3	-1.0	Kasp3B(Exome)_3	wsnp_Ku_c18538_27857915	3.15
QTwt.crc-3B	Twt_GLE99	3B	70.4	70.1–72.9	7.3	15.3	-1.0	Tdurum_contig27495_111	Kasp3B(survey)_17	3.08
QTwt.crc-3B	Twt_MOR99	3B	70.8	70.1–73.1	13.8	17.0	-1.0	Kasp3B(survey)_17	wsnp_Ex_c16378_24870688	3.06
QTwt.crc-3B	Twt_GLE00	3B	72.1	70.2–73.1	13.7	15.4	-1.0	Kasp3B(survey)_17	wsnp_Ex_c16378_24870688	3.02
QTwt.crc-3B	Twt_MOR00	3B	72.7	70.8–73.1	12.7	15.1	-1.0	Kasp3B(survey)_17	wsnp_Ex_c16378_24870688	3.03
QTwt.crc-3D	Twt_GLE00	3D.2	77.1	66.5–77.7	5.1	5.1	-0.6	barc270	RAC875_c5606_501	3.02
QTwt.crc-3D	Twt_MOR00	3D.2	77.1	67.0–77.7	3.8	4.0	-0.5	barc270	RAC875_c5606_501	3.03
QTwt.crc-3D	Twt_MOR99	3D.2	77.7	77.1–82.5	5.4	5.6	-0.6	RAC875_c5606_501	CAP7_c4219_359	3.06
QTwt.crc-4D	Twt_MOR99	4D	34.2	31.1–38.2	17.0	22.3	1.2	wmc617c	wMAS000002	3.06
QTwt.crc-4D	Twt_GLE00	4D	35	32.2–38.8	19.2	24.0	1.2	wMAS000002	wmc48b	3.02
QTwt.crc-4D	Twt_GLE99	4D	35.4	28.7–41.4	8.8	19.8	1.2	wMAS000002	wmc48b	3.08
QTwt.crc-4D	Twt_MOR00	4D	36.4	32.7–41.2	16.8	22.5	1.2	wMAS000002	wmc48b	3.03
QTwt.crc-4D	Twt_BRA04	4D	36.7	34.4–40.3	28.8	31.0	1.8	wMAS000002	wmc48b	3.15
QTwt.crc-7A	Twt_MOR99	7A	84.1	83.5–85.2	5.2	5.4	0.6	Kukri_c53682_85	BS00103846_51	3.06
QTwt.crc-7A	Twt_GLE00	7A	84.1	83.5–85.2	3.3	3.2	0.5	Kukri_c53682_85	BS00103846_51	3.02
**Flour yield (Fyd)**										
QFyd.crc-1B	Fyd_99	1B.1	81.7	81.1–88.1	3.8	6.1	-0.6	BS00110231_51	gwm274a	3.14
QFyd.crc-1B	Fyd_avg	1B.1	92.1	90.9–93.0	5.1	6.0	-0.4	Excalibur_c37496_271	wPt-2257	3.07
QFyd.crc-3B	Fyd_00	3B	62.9	61.8–63.9	9.7	17.5	0.7	wsnp_Ku_c18538_27857915	wsnp_Ex_c4769_8510104	3.12
QFyd.crc-3B	Fyd_avg	3B	65.1	64.6–65.6	11.9	15.2	0.6	Kukri_c4310_489	TA002966-0294	3.07
QFyd.crc-3B	Fyd_99	3B	65.1	64.5–65.6	4.5	7.3	0.6	Kukri_c4310_489	TA002966-0294	3.14
QFyd.crc-3D	Fyd_avg	3D.2	77.2	77.1–82.0	4.9	5.7	-0.4	RAC875_c5606_501	CAP7_c4219_359	3.07
QFyd.crc-3D	Fyd_00	3D.2	77.7	61.6–77.7	3.3	5.4	-0.4	RAC875_c5606_501	CAP7_c4219_359	3.12
QFyd.crc-4B	Fyd_avg	4B	53	52.4–53.5	3.9	4.5	-0.3	TA003708-0300	BS00066282_51	3.07
QFyd.crc-4B	Fyd_00	4B	54.2	53.5–54.8	3.1	5.0	-0.4	BS00066282_51	wmc657	3.12
QFyd.crc-7D	Fyd_avg	7D.2	13.8	13.7–14.3	16.3	21.7	0.7	wsnp_Ra_c6894_11980338	Excalibur_c22419_460	3.07
QFyd.crc-7D	Fyd_99	7D.2	15	13.7–18.9	11.3	20.7	1.0	Excalibur_c22419_460	wsnp_CAP8_rep_c9647_4198594	3.14
QFyd.crc-7D	Fyd_00	7D.2	23.9	22.0–28.1	8.7	16.0	0.6	wsnp_CAP8_rep_c9647_4198594	Kukri_c35508_426	3.12
QFyd.crc-7D	Fyd_98	7D.2	43.3	42.4–44.6	3.9	13.1	0.7	Ku_c26916_669	wsnp_Ex_c11813_18968198	3.11

^a^ BRA = Brandon, GLE = Glenlea, MOR = Morden, 98 = 1998, 99 = 1999, 00 = 2000, 04 = 2004.

^b^ Confidence interval determined by one LOD drop-off.

^c^ Additive effect of allele substitution. The units are those of the respective trait. A positive sign indicated that the ‘AC Domain’ allele increased the respective quantitative trait, and vice-versa.

**Table 3:** Inclusive Composite Interval Mapping (QIC) of QTL for 14 grain shape traits in the RL4452/‘AC Domain’ DH population from replicated trials.

QTL	Trait name^a^	Chr	Peak (cM)	CI (cM)^b^	LOD	r^2^ (%)	Add^c^	Left marker	Right marker	LOD threshold (α_0.05_)
QDMen.crc-1A	DMenG00A	1A	0	0–5.3	4.41	3.7	-0.445	Tdurum_contig42405_197	Tdurum_contig46413_779	3.0
QPer.crc-1A	Per2YRA	1A	0	0–5.9	3.98	4.9	-2.0269	Tdurum_contig42405_197	Tdurum_contig46413_779	3.0
QSzLn.crc-1A	SzLn2YRA	1A	0.3	0–7.8	4.92	5.3	-0.9539	Tdurum_contig42405_197	Tdurum_contig46413_779	3.1
QArPe.crc-1A	ArPeG00A	1A	1.9	0–8.8	4.86	3.8	-0.1095	Tdurum_contig42405_197	Tdurum_contig46413_779	3.1
QDMin.crc-1A	DMinG00A	1A	1.9	0–9.5	3.32	2.3	-0.315	Tdurum_contig42405_197	Tdurum_contig46413_779	3.1
QArPe.crc-1A	ArPe2YRA	1A	23.5	22.9–28.2	3.37	2.2	-0.0859	Excalibur_c3941_537	RAC875_c14926_589	3.1
QSzWd.crc-1A	SzWd2YRA	1A	23.5	22.9–27.1	3.46	2.4	-0.3309	Excalibur_c3941_537	RAC875_c14926_589	3.1
QSzWd.crc-1A	SzWdG00A	1A	23.5	22.9–27.3	3.48	2.5	-0.3159	Excalibur_c3941_537	RAC875_c14926_589	3.0
QArPe(var).crc-1B	ArPeG00S	1B.1	57.7	55.0–58.8	3.46	7.4	-0.0237	wsnp_Ra_c4296_7819139	RAC875_c8849_134	3.0
QArea(var).crc-1B	AreaG00S	1B.1	65.6	65.0–66.9	3.18	5.0	-10.3696	RAC875_c16391_426	CAP8_c818_370	3.0
QDMen.crc-1B	DMenB04A	1B.1	66.9	65.5–67.4	3.10	2.4	-0.4475	RAC875_c16391_426	CAP8_c818_370	3.0
QAMaL.crc-1D	AMaLG00A	1D	79.3	77.9–80.3	3.35	3.7	0.7464	Excalibur_c33661_412	BS00038418_51	3.1
QDMax.crc-1D	DMaxG00A	1D	79.3	77.9–80.3	4.35	5.2	0.9503	Excalibur_c33661_412	BS00038418_51	3.1
QSzLn.crc-1D	SzLn2YRA	1D	79.3	77.9–80.3	5.12	5.5	0.97	Excalibur_c33661_412	BS00038418_51	3.1
QPer.crc-1D	PerG00A	1D	81.1	79.2–90.5	4.85	5.6	2.1005	BS00038418_51	IAAV724	3.1
QSzLn.crc-1D	SzLnG00A	1D	87.4	80.3–96.0	5.11	7.1	1.0973	BS00038418_51	IAAV724	3.1
QAsym.crc-1D	AsymB04A	1D	97.1	96.1–97.5	4.03	5.4	1.6713	IAAV724	gpw0360	3.0
QAMaL.crc-1D	AMaLB04A	1D	97.5	96.1–107.1	3.97	4.1	0.8513	IAAV724	gpw0360	3.1
QDMax.crc-1D	DMax2YRA	1D	97.6	96.1–107.4	5.04	5.3	0.9635	gpw0360	BS00022188_51	3.2
QDMax.crc-1D	DMaxB04A	1D	97.6	96.1–107.9	3.71	4.9	0.9605	gpw0360	BS00022188_51	3.1
QSzLn.crc-1D	SzLnB04A	1D	97.6	96.1–108.0	3.74	5.0	0.9711	gpw0360	BS00022188_51	3.0
QAMaL.crc-1D	AMaL2YRA	1D	98.1	96.1–107.4	5.34	5.3	0.9239	gpw0360	BS00022188_51	3.1
QAsym.crc-1D	AsymG00A	1D	99.4	96.1–111.2	5.49	6.4	1.8218	gpw0360	BS00022188_51	3.1
QAsym(var).crc-1D	Asym2YRS	1D	99.5	96.1–113.7	4.59	6.8	0.4098	gpw0360	BS00022188_51	3.1
QPer.crc-1D	PerB04A	1D	99.6	96.1–109.8	3.83	4.1	1.9833	gpw0360	BS00022188_51	3.1
QAsym.crc-1D	Asym2YRA	1D	101.0	96.1–110.7	6.24	7.1	1.9083	gpw0360	BS00022188_51	3.0
QAsym(var).crc-1D	AsymB04S	1D	102.3	96.1–119.1	3.33	2.0	0.3884	gpw0360	BS00022188_51	3.1
QAMiL.crc-2B.1	AMiLG00A	2B.1	36.2	31.0–36.8	6.97	4.8	-0.4397	gpw5229	Excalibur_c40567_1893	3.1
QArPe.crc-2B.1	ArPe2YRA	2B.1	36.2	30.6–36.8	6.50	4.4	-0.1211	gpw5229	Excalibur_c40567_1893	3.1
QArPe.crc-2B.1	ArPeG00A	2B.1	36.2	31.8–36.8	6.42	4.8	-0.1225	gpw5229	Excalibur_c40567_1893	3.1
QDMen.crc-2B.1	DMenG00A	2B.1	36.2	28.5–36.8	4.11	3.5	-0.4282	gpw5229	Excalibur_c40567_1893	3.0
QDMin.crc-2B.1	DMinG00A	2B.1	36.2	28.4–36.8	6.29	4.1	-0.4216	gpw5229	Excalibur_c40567_1893	3.1
QRect(var).crc-2B.1	Rect2YRS	2B.1	36.2	31.9–36.8	6.34	6.6	0.0017	gpw5229	Excalibur_c40567_1893	3.0
QRect(var).crc-2B.1	RectB04S	2B.1	36.2	32.7–36.8	7.71	8.6	0.0022	gpw5229	Excalibur_c40567_1893	3.2
QRndn(var).crc-2B	Rndn2YRS	2B.1	36.2	28.2–36.8	3.20	4.1	0.0023	gpw5229	Excalibur_c40567_1893	3.1
QRndn(var).crc-2B	RndnB04S	2B.1	36.2	30.7–36.8	5.05	6.7	0.0035	gpw5229	Excalibur_c40567_1893	3.1
QSzWd.crc-2B.1	SzWdG00A	2B.1	36.2	31.3–36.8	6.52	5.0	-0.4394	gpw5229	Excalibur_c40567_1893	3.0
QSzWd.crc-2B.1	SzWdB04A	2B.1	36.2	31.7–36.8	14.09	9.7	-0.8567	gpw5229	Excalibur_c40567_1893	3.1
QSzWd.crc-2B.1	SzWd2YRA	2B.1	37.4	36.7–41.2	7.13	5.3	-0.4846	wsnp_Ex_rep_c68623_67474885	GENE-1999_98	3.1
QAMiL.crc-2B.1	AMiL2YRA	2B.1	38.2	36.7–41.5	6.38	3.9	-0.4388	wsnp_Ex_rep_c68623_67474885	GENE-1999_98	3.1
QAMiL.crc-2B.1	AMiLB04A	2B.1	38.6	36.7–41.1	14.53	11.9	-0.9161	wsnp_Ex_rep_c68623_67474885	GENE-1999_98	3.0
QDMin.crc-2B.1	DMinB04A	2B.1	39.0	36.7–41.2	16.11	11.6	-0.9374	GENE-1999_98	wPt-8404	3.1
QRect.crc-2B	Rect2YRA	2B.1	61.4	58.8–63.1	8.25	13.9	-0.0035	wsnp_Ku_c12721_20478606	Tdurum_contig54704_176	3.0
QRndn.crc-2B	Rndn2YRA	2B.1	61.6	58.8–63.1	8.53	15.0	0.0113	wsnp_Ku_c12721_20478606	Tdurum_contig54704_176	3.1
QSphr.crc-2B	Sphr2YRA	2B.1	61.9	58.9–63.1	7.68	12.4	-0.0063	wsnp_Ku_c12721_20478606	Tdurum_contig54704_176	3.0
QArPe.crc-2B.2	ArPeB04A	2B.1	63.2	60.2–64.4	9.04	7.4	-0.1876	Tdurum_contig54704_176	wsnp_Ex_c21092_30220702	3.0
QDMen.crc-2B.2	DMenB04A	2B.1	63.2	60.0–64.4	6.13	5.0	-0.64	Tdurum_contig54704_176	wsnp_Ex_c21092_30220702	3.0
QRect(var).crc-2B.2	RectG00S	2B.1	63.2	59.2–64.4	4.74	6.2	0.0016	Tdurum_contig54704_176	wsnp_Ex_c21092_30220702	3.1
QRect.crc-2B	RectB04A	2B.1	63.6	63.2–64.4	8.80	12.3	-0.0045	Tdurum_contig54704_176	wsnp_Ex_c21092_30220702	3.1
QSphr.crc-2B	SphrB04A	2B.1	63.9	63.2–64.4	8.80	11.9	-0.0085	Tdurum_contig54704_176	wsnp_Ex_c21092_30220702	3.2
QRect.crc-2B	RectG00A	2B.1	64.4	63.2–64.4	11.11	15.6	-0.0039	Tdurum_contig54704_176	wsnp_Ex_c21092_30220702	3.2
QRndn.crc-2B	RndnG00A	2B.1	64.4	59.8–64.4	5.55	7.5	0.0088	Tdurum_contig54704_176	wsnp_Ex_c21092_30220702	3.2
QRndn.crc-2B	RndnB04A	2B.1	64.4	63.2–64.4	9.63	11.9	0.0136	Tdurum_contig54704_176	wsnp_Ex_c21092_30220702	3.0
QSphr.crc-2B	SphrG00A	2B.1	64.4	63.2–64.4	6.76	9.8	-0.0054	Tdurum_contig54704_176	wsnp_Ex_c21092_30220702	3.1
QAMiL.crc-2B.2	AMiL2YRA	2B.1	70.0	69.5–70.6	5.11	3.0	-0.3877	BS00030497_51	Tdurum_contig62852_538	3.1
QArPe.crc-2B.2	ArPe2YRA	2B.1	70.0	69.5–70.6	5.22	3.4	-0.1075	BS00030497_51	Tdurum_contig62852_538	3.1
QSzWd.crc-2B.2	SzWd2YRA	2B.1	70.0	69.5–70.6	4.45	3.2	-0.375	BS00030497_51	Tdurum_contig62852_538	3.1
QAMiL.crc-2B.2	AMiLG00A	2B.1	70.1	69.5–70.6	7.22	5.0	-0.4483	Tdurum_contig62852_538	RAC875_c55059_202	3.1
QDMin.crc-2B.2	DMin2YRA	2B.1	70.1	69.5–70.6	10.74	9.7	-0.695	Tdurum_contig62852_538	RAC875_c55059_202	2.9
QDMin.crc-2B.2	DMinG00A	2B.1	70.1	69.5–70.6	8.29	5.6	-0.4908	Tdurum_contig62852_538	RAC875_c55059_202	3.1
QSzWd.crc-2B.2	SzWdG00A	2B.1	70.1	69.5–70.6	4.61	3.4	-0.3647	Tdurum_contig62852_538	RAC875_c55059_202	3.0
QArPe.crc-2B.2	ArPeG00A	2B.1	80.1	78.6–81.0	7.55	5.7	-0.1342	RFL_Contig1953_583	wsnp_CAP11_c114_140053	3.1
QArea.crc-2B	Area2YRA	2B.1	87.9	86.7–90.6	5.08	5.3	-60.0369	wsnp_Ex_rep_c105551_89940311	TA001450-1081	3.0
QDMen.crc-2B.3	DMen2YRA	2B.1	87.9	87.8–91.7	5.63	5.6	-0.5817	wsnp_Ex_rep_c105551_89940311	TA001450-1081	3.1
QDMen.crc-2B.3	DMenG00A	2B.1	87.9	86.7–90.6	5.26	4.5	-0.4883	wsnp_Ex_rep_c105551_89940311	TA001450-1081	3.0
QPer.crc-2B	Per2YRA	2B.1	87.9	86.7–90.6	3.70	4.6	-1.947	wsnp_Ex_rep_c105551_89940311	TA001450-1081	3.0
QArea.crc-2B	AreaB04A	2B.1	90.6	87.8–91.7	3.77	3.6	-57.0823	wsnp_Ex_rep_c105551_89940311	TA001450-1081	3.0
QArea.crc-2B	AreaG00A	2B.1	95.8	94.2–96.0	5.26	5.5	-56.4305	wmc500b	wsnp_Ex_c9729_16071358	3.1
QRect.crc-2D	RectG00A	2D	82.1	79.9–83.8	4.35	5.7	0.0023	gpw0163	BobWhite_c39793_88	3.2
QRndn.crc-2D	RndnG00A	2D	87.5	86.6–88.8	7.51	10.5	-0.0104	wPt-6847	gpw5256	3.2
QRndn.crc-2D	Rndn2YRA	2D	87.9	86.6–88.8	4.57	7.5	-0.008	wPt-6847	gpw5256	3.1
QRndn.crc-2D	RndnB04A	2D	87.9	86.6–88.8	3.17	3.7	-0.0076	wPt-6847	gpw5256	3.0
QRect.crc-2D	Rect2YRA	2D	88.0	86.6–88.8	4.52	7.1	0.0025	wPt-6847	gpw5256	3.0
QSphr.crc-2D	SphrG00A	2D	88.0	86.6–88.8	4.38	6.3	0.0043	wPt-6847	gpw5256	3.1
QSphr.crc-2D	SphrB04A	2D	88.0	86.6–88.8	3.24	4.2	0.005	wPt-6847	gpw5256	3.2
QSphr.crc-2D	Sphr2YRA	2D	88.1	86.6–88.8	4.94	7.6	0.0049	wPt-6847	gpw5256	3.0
QAMiL(var).crc-2D	AMiL2YRS	2D	101.6	100.3–102.6	4.05	6.1	0.0767	IACX14755	wsnp_Ku_c498_1036380	3.2
QAMiL(var).crc-2D	AMiLB04S	2D	101.7	100.3–102.6	3.69	8.1	0.1051	IACX14755	wsnp_Ku_c498_1036380	3.1
QDMin(var).crc-2D	DMinB04S	2D	101.9	100.9–104.3	3.27	6.4	0.0976	IACX14755	wsnp_Ku_c498_1036380	3.1
QSzWd(var).crc-2D	SzWdB04S	2D	101.9	100.3–102.6	3.33	6.6	0.0954	IACX14755	wsnp_Ku_c498_1036380	3.2
QSphr(var).crc-3A	Sphr2YRS	3A	65.9	64.6–68.3	5.01	12.6	0.0013	Excalibur_c2578_1966	wsnp_Ku_rep_c71761_71496470	3.0
QSphr(var).crc-3A	SphrB04S	3A	65.9	64.6–68.9	4.08	10.5	0.0016	Excalibur_c2578_1966	wsnp_Ku_rep_c71761_71496470	3.0
QSphr(var).crc-3A	SphrG00S	3A	65.9	64.6–67.9	5.40	13.7	0.0012	Excalibur_c2578_1966	wsnp_Ku_rep_c71761_71496470	3.2
QSzWd.crc-3B	SzWd2YRA	3B	0.1	0–0.6	3.13	2.2	-0.3152	Tdurum_contig50954_1393	Kukri_c15654_309	3.1
QArPe.crc-3B	ArPe2YRA	3B	0.2	0–0.6	5.87	4.0	-0.1165	Tdurum_contig50954_1393	Kukri_c15654_309	3.1
QPer.crc-3B	PerG00A	3B	0.2	0–0.6	4.77	5.4	-2.0615	Tdurum_contig50954_1393	Kukri_c15654_309	3.1
QAMiL.crc-3B	AMiL2YRA	3B	0.3	0–0.6	4.85	3.0	-0.3836	Tdurum_contig50954_1393	Kukri_c15654_309	3.1
QAMiL.crc-3B	AMiLG00A	3B	0.3	0–0.6	8.62	6.2	-0.5011	Tdurum_contig50954_1393	Kukri_c15654_309	3.1
QArea.crc-3B	AreaG00A	3B	0.3	0–0.6	6.25	6.8	-62.9258	Tdurum_contig50954_1393	Kukri_c15654_309	3.1
QArPe.crc-3B	ArPeG00A	3B	0.3	0–0.6	5.56	4.2	-0.1146	Tdurum_contig50954_1393	Kukri_c15654_309	3.1
QDMen.crc-3B	DMenG00A	3B	0.3	0–0.6	5.40	4.8	-0.5006	Tdurum_contig50954_1393	Kukri_c15654_309	3.0
QDMin.crc-3B	DMinG00A	3B	0.3	0–0.6	4.97	3.3	-0.3772	Tdurum_contig50954_1393	Kukri_c15654_309	3.1
QSzWd.crc-3B	SzWdG00A	3B	0.3	0–0.6	7.03	5.5	-0.464	Tdurum_contig50954_1393	Kukri_c15654_309	3.0
QAsym.crc-3B	AsymB04A	3B	65.6	65.1–66.2	6.66	9.2	2.1778	TA002966-0294	BS00078127_51	3.0
QArea(var).crc-3B	Area2YRS	3B	65.7	65.1–66.2	3.83	6.3	11.7414	BS00078127_51	TA001464-0572	3.0
QArea(var).crc-3B	AreaG00S	3B	65.7	65.1–66.2	5.17	8.4	13.4493	BS00078127_51	TA001464-0572	3.0
QAsym.crc-3B	Asym2YRA	3B	65.7	65.1–66.2	10.96	11.8	2.4649	BS00078127_51	TA001464-0572	3.0
QAsym.crc-3B	AsymG00A	3B	65.7	65.1–66.2	9.11	10.5	2.3461	BS00078127_51	TA001464-0572	3.1
QAsym(var).crc-3B	AsymB04S	3B	68.4	67.3–69.0	8.51	4.8	0.6076	Excalibur_c73633_120	wsnp_Ex_rep_c69664_68618163	3.1
QPer(var).crc-3B	PerG00S	3B	68.4	67.3–69.0	3.35	6.9	0.3687	Excalibur_c73633_120	wsnp_Ex_rep_c69664_68618163	3.1
QAsym(var).crc-3B	Asym2YRS	3B	70.2	70.1–70.7	7.88	11.5	0.5337	Tdurum_contig27495_111	Kasp3B(survey)_17	3.1
QDMen(var).crc-3B	DMenG00S	3B	70.2	70.1–70.7	4.82	10.0	0.117	Tdurum_contig27495_111	Kasp3B(survey)_17	3.2
QAsym(var).crc-3B	AsymG00S	3B	72.5	70.1–73.1	8.41	17.1	0.6415	Kasp3B(survey)_17	wsnp_Ex_c16378_24870688	3.1
QAMiL(var).crc-3B	AMiLG00S	3B	74.5	73.1–77.9	4.34	13.4	0.099	wsnp_Ex_c37115_44930934	wsnp_Ex_c18915_27811736	3.1
QArPe(var).crc-3B	ArPeG00S	3B	74.5	73.1–78.0	4.34	9.3	0.0266	wsnp_Ex_c37115_44930934	wsnp_Ex_c18915_27811736	3.0
QDMin(var).crc-3B	DMinG00S	3B	74.5	73.1–77.5	4.66	14.7	0.1055	wsnp_Ex_c37115_44930934	wsnp_Ex_c18915_27811736	3.0
QSzWd(var).crc-3B	SzWdG00S	3B	74.5	73.1–77.8	4.62	14.0	0.1038	wsnp_Ex_c37115_44930934	wsnp_Ex_c18915_27811736	3.0
QArPe.crc-3D	ArPe2YRA	3D.2	2.3	0.5–3.7	3.39	2.2	-0.0855	wsnp_Ra_c17636_26538543	gwm191a	3.1
QAMiL.crc-3D	AMiLG00A	3D.2	14.1	12.4–25.8	5.33	3.6	-0.3799	BobWhite_c23305_1192	wmc552	3.1
QSzWd.crc-3D	SzWdG00A	3D.2	14.1	12.4–27.4	4.52	3.3	-0.3605	BobWhite_c23305_1192	wmc552	3.0
QDMin.crc-3D	DMinG00A	3D.2	15.7	12.4–29.3	6.56	4.5	-0.4407	BobWhite_c23305_1192	wmc552	3.1
QArPe.crc-3D	ArPeG00A	3D.2	21.1	14.0–32.4	6.26	5.7	-0.1344	BobWhite_c23305_1192	wmc552	3.1
QArea.crc-3D	AreaG00A	3D.2	22.4	12.4–22.4	3.45	4.4	-50.6042	BobWhite_c23305_1192	wmc552	3.1
QDMen.crc-3D	DMenG00A	3D.2	24.8	14.0–35.4	7.20	8.3	-0.659	BobWhite_c23305_1192	wmc552	3.0
QSzLn.crc-3D	SzLnG00A	3D.2	34.3	21.7–48.4	4.38	7.9	-1.1569	BobWhite_c23305_1192	wmc552	3.1
QSzLn.crc-3D	SzLn2YRA	3D.2	35.0	20.4–48.9	4.09	5.8	-0.9973	BobWhite_c23305_1192	wmc552	3.1
QPer.crc-3D	PerG00A	3D.2	35.5	24.0–48.3	5.19	8.1	-2.5149	BobWhite_c23305_1192	wmc552	3.1
QAMaL.crc-3D	AMaL2YRA	3D.2	78.5	77.7–84.0	4.24	4.0	-0.8077	CAP7_c4219_359	wsnp_Ex_c12369_19730765	3.1
QDMax.crc-3D	DMaxG00A	3D.2	80.4	77.7–84.5	4.07	5.1	-0.9385	CAP7_c4219_359	wsnp_Ex_c12369_19730765	3.1
QDMax.crc-3D	DMax2YRA	3D.2	80.7	77.7–84.3	3.73	4.0	-0.8408	CAP7_c4219_359	wsnp_Ex_c12369_19730765	3.2
QAMaL.crc-3D	AMaLG00A	3D.2	81.0	77.7–84.5	5.39	6.3	-0.9763	CAP7_c4219_359	wsnp_Ex_c12369_19730765	3.1
QAMaL.crc-3D	AMaLB04A	3D.2	82.8	77.7–84.7	3.55	3.7	-0.8024	CAP7_c4219_359	wsnp_Ex_c12369_19730765	3.1
QSzLn.crc-4A.1	SzLn2YRA	4A	37.4	36.6–38.3	7.54	8.4	1.2002	wsnp_Ex_c5492_9691241	wsnp_Ex_rep_c66706_65037564	3.1
QAMaL.crc-4A.1	AMaL2YRA	4A	37.7	36.6–38.3	9.53	9.8	1.2573	wsnp_Ex_c5492_9691241	wsnp_Ex_rep_c66706_65037564	3.1
QAMaL.crc-4A.1	AMaLG00A	4A	37.7	36.6–38.3	7.18	8.2	1.1231	wsnp_Ex_c5492_9691241	wsnp_Ex_rep_c66706_65037564	3.1
QAMaL.crc-4A.1	AMaLB04A	4A	37.7	36.6–38.3	9.51	10.7	1.3735	wsnp_Ex_c5492_9691241	wsnp_Ex_rep_c66706_65037564	3.1
QAsym.crc-4A.1	Asym2YRA	4A	37.7	36.6–38.3	4.14	4.1	1.449	wsnp_Ex_c5492_9691241	wsnp_Ex_rep_c66706_65037564	3.0
QAsym.crc-4A.1	AsymB04A	4A	37.7	36.6–38.3	5.18	7.0	1.9068	wsnp_Ex_c5492_9691241	wsnp_Ex_rep_c66706_65037564	3.0
QDMax.crc-4A.1	DMax2YRA	4A	37.7	36.6–38.3	7.73	8.4	1.2189	wsnp_Ex_c5492_9691241	wsnp_Ex_rep_c66706_65037564	3.2
QDMax.crc-4A.1	DMaxG00A	4A	37.7	36.6–38.3	7.57	9.5	1.2824	wsnp_Ex_c5492_9691241	wsnp_Ex_rep_c66706_65037564	3.1
QDMax.crc-4A.1	DMaxB04A	4A	37.7	36.6–38.3	8.82	12.5	1.5386	wsnp_Ex_c5492_9691241	wsnp_Ex_rep_c66706_65037564	3.1
QPer.crc-4A.1	PerG00A	4A	37.7	36.6–38.3	5.72	6.5	2.2612	wsnp_Ex_c5492_9691241	wsnp_Ex_rep_c66706_65037564	3.1
QPer.crc-4A.1	PerB04A	4A	37.7	36.6–38.3	6.94	6.9	2.5781	wsnp_Ex_c5492_9691241	wsnp_Ex_rep_c66706_65037564	3.1
QSzLn.crc-4A.1	SzLnG00A	4A	37.7	36.6–38.3	6.34	8.3	1.1953	wsnp_Ex_c5492_9691241	wsnp_Ex_rep_c66706_65037564	3.1
QSzLn.crc-4A.1	SzLnB04A	4A	37.7	36.6–38.3	8.75	12.4	1.5407	wsnp_Ex_c5492_9691241	wsnp_Ex_rep_c66706_65037564	3.0
QRect.crc-4A	RectG00A	4A	38.8	38.2–39.4	4.80	6.3	-0.0025	wsnp_Ku_c13640_21686670	wsnp_Ex_c829_1621908	3.2
QRect.crc-4A	Rect2YRA	4A	39.3	38.8–40.9	5.66	8.8	-0.0028	wsnp_Ex_c829_1621908	Kukri_rep_c69389_1215	3.0
QRect.crc-4A	RectB04A	4A	39.3	38.8–40.9	5.11	6.7	-0.0034	wsnp_Ex_c829_1621908	Kukri_rep_c69389_1215	3.1
QRndn.crc-4A	Rndn2YRA	4A	39.3	38.8–40.9	4.80	7.7	0.0081	wsnp_Ex_c829_1621908	Kukri_rep_c69389_1215	3.1
QRndn.crc-4A	RndnB04A	4A	39.3	38.8–40.9	4.43	5.1	0.009	wsnp_Ex_c829_1621908	Kukri_rep_c69389_1215	3.0
QSphr.crc-4A	Sphr2YRA	4A	39.3	38.8–40.9	5.83	8.8	-0.0054	wsnp_Ex_c829_1621908	Kukri_rep_c69389_1215	3.0
QSphr.crc-4A	SphrG00A	4A	39.3	38.8–40.9	4.54	6.4	-0.0044	wsnp_Ex_c829_1621908	Kukri_rep_c69389_1215	3.1
QSphr.crc-4A	SphrB04A	4A	39.3	38.8–40.9	5.17	6.6	-0.0064	wsnp_Ex_c829_1621908	Kukri_rep_c69389_1215	3.2
QAMiL.crc-4A.1	AMiLG00A	4A	61.6	60.3–62.6	4.06	2.7	-0.3287	RAC875_c25124_182	wsnp_Ku_c4924_8816643	3.1
QDMin.crc-4A.1	DMinG00A	4A	61.6	60.3–62.6	5.86	3.8	-0.4053	RAC875_c25124_182	wsnp_Ku_c4924_8816643	3.1
QArea(var).crc-4A.1	AreaG00S	4A	63.8	63.2–65.9	4.76	7.7	12.8161	Tdurum_contig13489_292	wsnp_JD_c38619_27992279	3.0
QArPe(var).crc-4A	ArPeG00S	4A	63.8	63.2–65.7	4.12	8.8	0.0258	Tdurum_contig13489_292	wsnp_JD_c38619_27992279	3.0
QDMen(var).crc-4A.1	DMenG00S	4A	63.8	63.2–65.9	3.88	8.1	0.1051	Tdurum_contig13489_292	wsnp_JD_c38619_27992279	3.2
QAMaL(var).crc-4A	AMaLB04S	4A	82.6	81.9–83.5	4.30	8.5	0.2175	RAC875_c88582_131	Excalibur_c74397_238	3.0
QAMaL(var).crc-4A	AMaL2YRS	4A	83.5	82.4–84.1	4.42	9.0	0.1885	RAC875_c88582_131	Excalibur_c74397_238	3.1
QAMaL.crc-4A.2	AMaL2YRA	4A	86.3	85.2–89.3	5.24	5.1	0.8999	RAC875_c7016_2039	Excalibur_c4325_1150	3.1
QAMaL.crc-4A.2	AMaLB04A	4A	86.3	86.2–90.1	7.09	7.7	1.1555	RAC875_c7016_2039	Excalibur_c4325_1150	3.1
QAsym.crc-4A.2	AsymG00A	4A	86.3	85.2–89.5	5.15	5.6	1.7105	RAC875_c7016_2039	Excalibur_c4325_1150	3.1
QDMax.crc-4A.2	DMax2YRA	4A	86.3	85.2–89.5	6.00	6.4	1.0552	RAC875_c7016_2039	Excalibur_c4325_1150	3.2
QAsym(var).crc-4A	Asym2YRS	4A	87.4	86.2–90.1	4.10	5.8	0.3782	RAC875_c7016_2039	Excalibur_c4325_1150	3.1
QPer.crc-4A.2	PerB04A	4A	87.7	86.2–90.1	8.63	8.9	2.9068	RAC875_c7016_2039	Excalibur_c4325_1150	3.1
QArea.crc-4A	AreaG00A	4A	89.6	86.2–90.1	3.71	3.8	46.7029	Excalibur_c4325_1150	RAC875_c59673_500	3.1
QDMax.crc-4A.2	DMaxB04A	4A	89.6	89.5–92.7	5.73	7.8	1.2042	Excalibur_c4325_1150	RAC875_c59673_500	3.1
QDMin.crc-4A.2	DMin2YRA	4A	89.6	86.2–90.1	3.25	2.6	0.3624	Excalibur_c4325_1150	RAC875_c59673_500	2.9
QSzLn.crc-4A.2	SzLnB04A	4A	89.6	89.5–92.7	5.69	7.7	1.2068	Excalibur_c4325_1150	RAC875_c59673_500	3.0
QAMiL.crc-4A.2	AMiL2YRA	4A	90.1	89.5–93.3	3.83	2.2	0.3329	Excalibur_c4325_1150	RAC875_c59673_500	3.1
QAMiL.crc-4A.2	AMiLG00A	4A	90.1	89.5–92.1	8.25	5.8	0.4827	Excalibur_c4325_1150	RAC875_c59673_500	3.1
QArea.crc-4A	Area2YRA	4A	90.1	86.2–90.1	6.60	7.0	69.0294	Excalibur_c4325_1150	RAC875_c59673_500	3.0
QArea.crc-4A	AreaB04A	4A	90.1	86.2–90.1	6.50	6.4	76.291	Excalibur_c4325_1150	RAC875_c59673_500	3.0
QArea(var).crc-4A.2	Area2YRS	4A	90.1	89.5–92.7	5.34	9.0	14.0057	Excalibur_c4325_1150	RAC875_c59673_500	3.0
QArPe.crc-4A	ArPe2YRA	4A	90.1	89.5–92.7	7.76	5.3	0.1335	Excalibur_c4325_1150	RAC875_c59673_500	3.1
QArPe.crc-4A	ArPeG00A	4A	90.1	89.5–92.5	4.78	3.5	0.1046	Excalibur_c4325_1150	RAC875_c59673_500	3.1
QAsym(var).crc-4A	AsymB04S	4A	90.1	89.5–90.1	29.63	22.9	1.321	Excalibur_c4325_1150	RAC875_c59673_500	3.1
QDMen.crc-4A	DMen2YRA	4A	90.1	86.3–90.1	5.54	5.6	0.5782	Excalibur_c4325_1150	RAC875_c59673_500	3.1
QDMen.crc-4A	DMenG00A	4A	90.1	89.5–92.3	6.55	5.7	0.5497	Excalibur_c4325_1150	RAC875_c59673_500	3.0
QDMen.crc-4A	DMenB04A	4A	90.1	89.5–93.0	8.22	6.9	0.753	Excalibur_c4325_1150	RAC875_c59673_500	3.0
QDMin.crc-4A.2	DMinG00A	4A	90.1	89.5–91.9	10.04	6.9	0.5473	Excalibur_c4325_1150	RAC875_c59673_500	3.1
QPer.crc-4A.2	Per2YRA	4A	90.1	86.2–90.1	7.87	10.3	2.918	Excalibur_c4325_1150	RAC875_c59673_500	3.0
QSzLn.crc-4A.2	SzLn2YRA	4A	90.1	86.2–90.1	4.83	5.1	0.9367	Excalibur_c4325_1150	RAC875_c59673_500	3.1
QSzWd.crc-4A	SzWd2YRA	4A	90.1	86.2–90.1	4.28	3.0	0.3672	Excalibur_c4325_1150	RAC875_c59673_500	3.1
QSzWd.crc-4A	SzWdG00A	4A	90.1	89.5–93.4	3.56	2.6	0.3179	Excalibur_c4325_1150	RAC875_c59673_500	3.0
QAMaL(var).crc-4A	AMaLG00S	4A	90.2	89.5–94.4	4.15	8.5	0.1867	RAC875_c59673_500	RFL_Contig4334_379	3.1
QArea(var).crc-4A.2	AreaB04S	4A	90.2	89.5–92.6	8.60	13.3	21.4436	RAC875_c59673_500	RFL_Contig4334_379	3.0
QArPe.crc-4A	ArPeB04A	4A	90.2	89.5–94.0	5.73	4.5	0.1462	RAC875_c59673_500	RFL_Contig4334_379	3.0
QDMen(var).crc-4A.2	DMenB04S	4A	90.2	89.5–93.6	3.37	7.2	0.1098	RAC875_c59673_500	RFL_Contig4334_379	3.1
QPer(var).crc-4A	Per2YRS	4A	90.2	89.5–93.4	4.79	9.3	0.428	RAC875_c59673_500	RFL_Contig4334_379	3.0
QPer(var).crc-4A	PerG00S	4A	90.2	89.5–94.3	3.56	7.3	0.3799	RAC875_c59673_500	RFL_Contig4334_379	3.1
QPer(var).crc-4A	PerB04S	4A	90.2	89.5–93.2	4.35	8.8	0.4581	RAC875_c59673_500	RFL_Contig4334_379	3.0
QSphr.crc-4B	SphrG00A	4B	38.4	33.2–42.8	7.32	11.3	0.0058	BS00022431_51	GENE-3024_59	3.1
QRect.crc-4B	Rect2YRA	4B	39.2	32.1–42.8	5.37	8.6	0.0028	BS00022431_51	GENE-3024_59	3.0
QRndn.crc-4B	RndnG00A	4B	39.4	34.1–43.3	8.02	11.9	-0.0112	BS00022431_51	GENE-3024_59	3.2
QSphr.crc-4B	Sphr2YRA	4B	39.6	33.0–42.8	6.02	9.4	0.0056	BS00022431_51	GENE-3024_59	3.0
QRect.crc-4B	RectG00A	4B	40.7	33.9–43.3	6.44	8.8	0.003	BS00022431_51	GENE-3024_59	3.2
QRndn.crc-4B	Rndn2YRA	4B	40.7	33.9–43.3	5.53	9.2	-0.009	BS00022431_51	GENE-3024_59	3.1
QAMaL.crc-4B	AMaLB04A	4B	43.3	40.0–43.3	12.97	15.2	-1.6474	GENE-3024_59	Excalibur_rep_c113261_400	3.1
QAsym.crc-4B	AsymB04A	4B	43.3	39.0–43.3	10.01	14.5	-2.7553	GENE-3024_59	Excalibur_rep_c113261_400	3.0
QAsym.crc-4B	Asym2YRA	4B	51.3	50.7–51.8	14.04	15.9	-2.8586	Tdurum_contig57516_269	BS00105308_51	3.0
QDMax.crc-4B	DMax2YRA	4B	51.3	49.3–51.3	15.15	18.2	-1.7894	Tdurum_contig57516_269	BS00105308_51	3.2
QSzLn.crc-4B	SzLn2YRA	4B	51.3	49.3–51.3	16.66	21.0	-1.8967	Tdurum_contig57516_269	BS00105308_51	3.1
QAMaL.crc-4B	AMaL2YRA	4B	52.4	51.8–52.9	16.04	18.1	-1.7029	Tdurum_contig29989_132	Tdurum_contig5562_441	3.1
QArea.crc-4B	AreaB04A	4B	52.4	51.9–52.9	15.18	16.9	-124.204	Tdurum_contig29989_132	Tdurum_contig5562_441	3.0
QArPe(var).crc-4B	ArPe2YRS	4B	52.4	51.8–52.9	5.89	13.9	-0.0291	Tdurum_contig29989_132	Tdurum_contig5562_441	3.1
QDMax.crc-4B	DMaxB04A	4B	52.4	51.8–52.9	11.05	16.2	-1.7438	Tdurum_contig29989_132	Tdurum_contig5562_441	3.1
QDMen(var).crc-4B	DMen2YRS	4B	52.4	51.8–52.9	6.82	15.9	-0.1314	Tdurum_contig29989_132	Tdurum_contig5562_441	3.1
QDMen(var).crc-4B	DMenG00S	4B	52.4	51.8–52.9	5.56	11.8	-0.1274	Tdurum_contig29989_132	Tdurum_contig5562_441	3.2
QPer.crc-4B	PerB04A	4B	52.4	51.8–52.9	15.25	17.2	-4.0413	Tdurum_contig29989_132	Tdurum_contig5562_441	3.1
QSzLn.crc-4B	SzLnB04A	4B	52.4	51.8–52.9	10.97	16.1	-1.7469	Tdurum_contig29989_132	Tdurum_contig5562_441	3.0
QAMaL.crc-4B	AMaLG00A	4B	52.5	52.4–53.5	15.12	19.4	-1.7161	Tdurum_contig5562_441	TA003708-0300	3.1
QArea.crc-4B	Area2YRA	4B	52.5	52.4–53.5	15.85	19.0	-114.4186	Tdurum_contig5562_441	TA003708-0300	3.0
QArea(var).crc-4B	Area2YRS	4B	52.5	52.4–53.5	13.04	24.4	-23.0986	Tdurum_contig5562_441	TA003708-0300	3.0
QAsym.crc-4B	AsymG00A	4B	52.5	52.4–53.5	14.87	18.5	-3.1098	Tdurum_contig5562_441	TA003708-0300	3.1
QAsym(var).crc-4B	AsymG00S	4B	52.5	52.4–53.5	9.11	18.7	-0.6711	Tdurum_contig5562_441	TA003708-0300	3.1
QDMax.crc-4B	DMaxG00A	4B	52.5	52.4–53.5	13.99	19.1	-1.8178	Tdurum_contig5562_441	TA003708-0300	3.1
QDMax(var).crc-4B	DMax2YRS	4B	52.5	51.8–52.9	6.32	13.3	-0.2212	Tdurum_contig5562_441	TA003708-0300	3.0
QDMax(var).crc-4B	DMaxB04S	4B	52.5	51.8–52.9	4.14	9.1	-0.2171	Tdurum_contig5562_441	TA003708-0300	3.1
QDMen.crc-4B	DMen2YRA	4B	52.5	52.4–53.5	13.91	15.8	-0.9733	Tdurum_contig5562_441	TA003708-0300	3.1
QDMin.crc-4B	DMin2YRA	4B	52.5	51.8–52.9	5.48	4.6	-0.4794	Tdurum_contig5562_441	TA003708-0300	2.9
QPer.crc-4B	Per2YRA	4B	52.5	52.4–53.5	16.36	23.9	-4.4629	Tdurum_contig5562_441	TA003708-0300	3.0
QPer(var).crc-4B	PerG00S	4B	52.5	52.4–53.5	6.56	14.1	-0.5268	Tdurum_contig5562_441	TA003708-0300	3.1
QSzLn(var).crc-4B	SzLn2YRS	4B	52.5	51.8–52.9	6.23	12.9	-0.2214	Tdurum_contig5562_441	TA003708-0300	3.1
QSzLn(var).crc-4B	SzLnB04S	4B	52.5	51.8–52.9	4.13	9.2	-0.2181	Tdurum_contig5562_441	TA003708-0300	3.0
QSzWd.crc-4B	SzWdB04A	4B	52.5	51.8–52.9	10.16	6.6	-0.709	Tdurum_contig5562_441	TA003708-0300	3.1
QAMaL(var).crc-4B	AMaL2YRS	4B	52.9	51.8–52.9	6.68	14.0	-0.2359	Tdurum_contig5562_441	TA003708-0300	3.1
QAMaL(var).crc-4B	AMaLG00S	4B	52.9	52.4–53.5	6.61	14.0	-0.2402	Tdurum_contig5562_441	TA003708-0300	3.1
QAMiL.crc-4B	AMiLG00A	4B	52.9	51.8–52.9	8.41	5.9	-0.4881	Tdurum_contig5562_441	TA003708-0300	3.1
QArPe.crc-4B	ArPeG00A	4B	52.9	51.8–52.9	13.21	10.8	-0.1846	Tdurum_contig5562_441	TA003708-0300	3.1
QArPe.crc-4B	ArPeB04A	4B	52.9	51.9–52.9	12.75	11.0	-0.2295	Tdurum_contig5562_441	TA003708-0300	3.0
QAsym(var).crc-4B	Asym2YRS	4B	52.9	51.8–52.9	12.54	19.6	-0.6993	Tdurum_contig5562_441	TA003708-0300	3.1
QDMax(var).crc-4B	DMaxG00S	4B	52.9	52.4–53.5	8.49	0.5	-0.2571	Tdurum_contig5562_441	TA003708-0300	3.1
QDMen.crc-4B	DMenB04A	4B	52.9	51.9–52.9	15.11	13.9	-1.0724	Tdurum_contig5562_441	TA003708-0300	3.0
QSzLn(var).crc-4B	SzLnG00S	4B	52.9	52.4–53.5	8.41	0.5	-0.257	Tdurum_contig5562_441	TA003708-0300	3.1
QSzWd.crc-4B	SzWdG00A	4B	52.9	51.8–52.9	10.36	8.3	-0.5679	Tdurum_contig5562_441	TA003708-0300	3.0
QArea(var).crc-4B	AreaB04S	4B	53.0	52.4–53.5	10.50	16.7	-24.0438	TA003708-0300	BS00066282_51	3.0
QPer(var).crc-4B	Per2YRS	4B	53.0	52.4–53.5	7.42	15.0	-0.5432	TA003708-0300	BS00066282_51	3.0
QAMiL.crc-4B	AMiL2YRA	4B	53.5	52.9–53.5	18.31	13.0	-0.8057	TA003708-0300	BS00066282_51	3.1
QArea.crc-4B	AreaG00A	4B	53.5	52.4–53.5	14.08	16.4	-97.877	TA003708-0300	BS00066282_51	3.1
QArea(var).crc-4B	AreaG00S	4B	53.5	52.4–53.5	12.09	21.6	-21.5565	TA003708-0300	BS00066282_51	3.0
QArPe.crc-4B	ArPe2YRA	4B	53.5	52.4–53.5	16.88	13.0	-0.2092	TA003708-0300	BS00066282_51	3.1
QArPe(var).crc-4B	ArPeG00S	4B	53.5	52.9–54.1	4.98	10.8	-0.0286	TA003708-0300	BS00066282_51	3.0
QDMen.crc-4B	DMenG00A	4B	53.5	52.4–53.5	16.35	16.3	-0.9274	TA003708-0300	BS00066282_51	3.0
QDMin.crc-4B	DMinB04A	4B	53.5	52.9–54.2	9.31	6.1	-0.6804	TA003708-0300	BS00066282_51	3.1
QPer.crc-4B	PerG00A	4B	53.5	53.0–53.5	15.70	20.5	-3.9994	TA003708-0300	BS00066282_51	3.1
QSzWd.crc-4B	SzWd2YRA	4B	53.5	52.4–53.5	11.77	9.2	-0.6402	TA003708-0300	BS00066282_51	3.1
QDMin.crc-4B	DMinG00A	4B	53.6	52.9–54.2	8.42	5.7	-0.4961	BS00066282_51	wmc657	3.1
QAMiL.crc-4B	AMiLB04A	4B	53.8	52.9–54.2	7.43	5.5	-0.6257	BS00066282_51	wmc657	3.0
QAMiL(var).crc-4B	AMiL2YRS	4B	54.1	53.5–54.8	6.25	9.1	-0.0942	BS00066282_51	wmc657	3.2
QDMin(var).crc-4B	DMin2YRS	4B	54.1	53.5–54.8	6.05	10.5	-0.0997	BS00066282_51	wmc657	3.0
QAMaL(var).crc-4B	AMaLB04S	4B	54.2	53.5–54.8	4.20	8.3	-0.2155	BS00066282_51	wmc657	3.0
QAMiL(var).crc-4B	AMiLB04S	4B	54.2	53.5–54.8	3.39	6.7	-0.0965	BS00066282_51	wmc657	3.1
QArPe(var).crc-4B	ArPeB04S	4B	54.2	53.5–54.8	4.97	11.1	-0.0327	BS00066282_51	wmc657	3.0
QAsym(var).crc-4B	AsymB04S	4B	54.2	53.5–54.8	10.73	6.3	-0.6943	BS00066282_51	wmc657	3.1
QDMen(var).crc-4B	DMenB04S	4B	54.2	53.5–54.8	5.55	12.2	-0.1433	BS00066282_51	wmc657	3.1
QDMin(var).crc-4B	DMinB04S	4B	54.2	53.5–54.8	4.88	8.9	-0.1158	BS00066282_51	wmc657	3.1
QPer(var).crc-4B	PerB04S	4B	54.2	53.5–54.8	4.93	10.0	-0.4902	BS00066282_51	wmc657	3.0
QSzWd(var).crc-4B	SzWd2YRS	4B	54.2	53.5–54.8	4.58	9.4	-0.0862	BS00066282_51	wmc657	3.1
QSzWd(var).crc-4B	SzWdB04S	4B	54.2	53.5–54.8	4.73	8.9	-0.111	BS00066282_51	wmc657	3.2
QAMiL.crc-4D	AMiL2YRA	4D	34.2	32.5–35.9	42.07	43.3	1.4647	wmc617c	wMAS000002	3.1
QAMiL.crc-4D	AMiLG00A	4D	34.2	31.9–37.5	33.42	34.1	1.17	wmc617c	wMAS000002	3.1
QAMiL.crc-4D	AMiLB04A	4D	34.2	31.9–36.8	36.68	41.5	1.7134	wmc617c	wMAS000002	3.0
QArea.crc-4D	AreaG00A	4D	34.2	27.5–38.7	11.89	13.5	88.5	wmc617c	wMAS000002	3.1
QArPe.crc-4D	ArPe2YRA	4D	34.2	32.2–36.3	34.59	35.1	0.3431	wmc617c	wMAS000002	3.1
QArPe.crc-4D	ArPeB04A	4D	34.2	31.9–36.3	30.82	34.8	0.4067	wmc617c	wMAS000002	3.0
QDMen.crc-4D	DMenB04A	4D	34.2	31.7–36.9	24.08	25.4	1.4441	wmc617c	wMAS000002	3.0
QDMin.crc-4D	DMinB04A	4D	34.2	32.1–37.3	41.12	43.3	1.8107	wmc617c	wMAS000002	3.1
QPer.crc-4D	PerB04A	4D	34.2	29.0–38.2	8.54	8.7	2.8765	wmc617c	wMAS000002	3.1
QSzWd.crc-4D	SzWd2YRA	4D	34.2	32.7–36.8	38.08	44.8	1.4058	wmc617c	wMAS000002	3.1
QSzWd.crc-4D	SzWdB04A	4D	34.2	32.2–36.7	41.49	43.1	1.8076	wmc617c	wMAS000002	3.1
QArea.crc-4D	AreaB04A	4D	34.3	31.5–38.0	19.07	22.5	142.9027	wMAS000002	wmc48b	3.0
QArPe.crc-4D	ArPeG00A	4D	34.3	32.3–38.5	27.96	28.4	0.299	wMAS000002	wmc48b	3.1
QDMen.crc-4D	DMenG00A	4D	34.3	31.3–39.3	16.34	16.5	0.9301	wMAS000002	wmc48b	3.0
QSzWd.crc-4D	SzWdG00A	4D	34.3	32.4–37.5	32.81	36.9	1.1957	wMAS000002	wmc48b	3.0
QDMin.crc-4D	DMinG00A	4D	34.4	32.6–37.9	35.05	35.2	1.2324	wMAS000002	wmc48b	3.1
QArea.crc-4D	Area2YRA	4D	34.6	31.7–39.6	14.87	17.9	110.5507	wMAS000002	wmc48b	3.0
QRect.crc-4D.1	RectG00A	4D	35.3	30.6–40.8	11.86	17.8	0.0041	wMAS000002	wmc48b	3.2
QDMin.crc-4D	DMin2YRA	4D	35.4	32.9–38.9	34.88	45.7	1.509	wMAS000002	wmc48b	2.9
QRect(var).crc-4D	Rect2YRS	4D	35.4	33.8–39.1	33.91	53.3	-0.0049	wMAS000002	wmc48b	3.0
QRndn.crc-4D.1	RndnG00A	4D	35.4	31.4–40.8	11.40	17.5	-0.0134	wMAS000002	wmc48b	3.2
QSphr.crc-4D.1	SphrG00A	4D	35.4	31.2–40.8	10.77	17.5	0.0071	wMAS000002	wmc48b	3.1
QDMen.crc-4D	DMen2YRA	4D	35.6	32.1–40.0	19.92	25.5	1.2353	wMAS000002	wmc48b	3.1
QSphr.crc-4D.1	Sphr2YRA	4D	35.7	31.9–40.5	16.44	30.1	0.0098	wMAS000002	wmc48b	3.0
QRect.crc-4D.1	Rect2YRA	4D	36.0	30.9–40.9	15.27	28.6	0.0051	wMAS000002	wmc48b	3.0
QRect(var).crc-4D	RectB04S	4D	36.0	34.2–39.9	31.88	51.0	-0.0053	wMAS000002	wmc48b	3.2
QRndn.crc-4D.1	Rndn2YRA	4D	36.1	31.0–41.5	13.84	26.5	-0.015	wMAS000002	wmc48b	3.1
QRect(var).crc-4D	RectG00S	4D	37.2	34.3–42.0	25.22	45.2	-0.0042	wMAS000002	wmc48b	3.1
QRndn.crc-4D.1	RndnB04A	4D	37.7	34.4–41.9	24.11	38.8	-0.0246	wMAS000002	wmc48b	3.0
QRndn(var).crc-4D	RndnG00S	4D	37.9	34.5–43.6	17.92	35.3	-0.006	wMAS000002	wmc48b	3.0
QRect.crc-4D.1	RectB04A	4D	38.1	34.7–42.6	21.43	37.3	0.0079	wMAS000002	wmc48b	3.1
QSphr.crc-4D.1	SphrB04A	4D	38.3	34.9–42.8	22.99	39.3	0.0154	wMAS000002	wmc48b	3.2
QRndn(var).crc-4D	Rndn2YRS	4D	39.3	35.6–43.9	24.34	45.0	-0.0075	wMAS000002	wmc48b	3.1
QRndn(var).crc-4D	RndnB04S	4D	39.9	36.0–44.4	23.49	43.3	-0.009	wMAS000002	wmc48b	3.1
QRndn.crc-4D.2	RndnG00A	4D	51.8	49.9–54.2	8.08	11.4	-0.0108	wmc48b	wsnp_BE444858D_Ta_1_1	3.2
QSphr.crc-4D.2	SphrG00A	4D	51.9	49.9–54.6	8.98	13.5	0.0063	wsnp_BE444858D_Ta_1_1	wsnp_Ex_c42133_48794975	3.1
QAsym(var).crc-4D	AsymB04S	4D	53.3	49.9–54.6	5.43	3.0	-0.4792	wsnp_BE444858D_Ta_1_1	wsnp_Ex_c42133_48794975	3.1
QAsym(var).crc-4D	Asym2YRS	4D	54.2	49.9–54.6	3.76	5.2	-0.3598	wsnp_BE444858D_Ta_1_1	wsnp_Ex_c42133_48794975	3.1
QRect.crc-4D.2	RectG00A	4D	54.4	49.9–54.6	7.42	10.0	0.0031	wsnp_BE444858D_Ta_1_1	wsnp_Ex_c42133_48794975	3.2
QAMiL.crc-5B.1	AMiLB04A	5B	56.2	55.5–57.2	9.97	7.6	0.7347	wsnp_Ex_rep_c66696_65023462	wsnp_RFL_Contig4565_5399994	3.0
QDMin.crc-5B.1	DMinB04A	5B	56.5	55.5–57.2	10.03	6.6	0.7086	wsnp_Ex_rep_c66696_65023462	wsnp_RFL_Contig4565_5399994	3.1
QAMiL.crc-5B.1	AMiL2YRA	5B	57.7	57.2–59.3	5.33	3.2	0.3963	BS00110635_51	TA004924-0669	3.1
QDMen.crc-5B	DMen2YRA	5B	57.8	57.2–59.3	4.92	4.9	0.5422	TA004924-0669	wsnp_Ku_c17875_27051169	3.1
QDMin.crc-5B.1	DMin2YRA	5B	57.8	57.2–59.3	3.52	2.9	0.3776	TA004924-0669	wsnp_Ku_c17875_27051169	2.9
QSzWd.crc-5B.1	SzWd2YRA	5B	59.0	57.7–59.9	4.13	2.9	0.3608	TA004924-0669	wsnp_Ku_c17875_27051169	3.1
QArPe.crc-5B.1	ArPe2YRA	5B	59.2	57.7–59.9	5.63	3.7	0.1119	TA004924-0669	wsnp_Ku_c17875_27051169	3.1
QArea.crc-5B	Area2YRA	5B	59.3	57.7–59.3	4.93	5.1	58.8311	TA004924-0669	wsnp_Ku_c17875_27051169	3.0
QArea.crc-5B	AreaB04A	5B	59.3	57.2–59.3	8.79	8.9	89.9434	TA004924-0669	wsnp_Ku_c17875_27051169	3.0
QArPe.crc-5B.1	ArPeB04A	5B	59.4	57.7–59.9	11.18	9.5	0.2119	wsnp_Ku_c17875_27051169	wsnp_Ex_c24933_34187952	3.0
QDMen.crc-5B	DMenB04A	5B	59.4	57.7–59.8	12.06	10.7	0.9362	wsnp_Ku_c17875_27051169	wsnp_Ex_c24933_34187952	3.0
QPer.crc-5B	PerB04A	5B	59.4	57.7–59.9	5.15	5.0	2.1802	wsnp_Ku_c17875_27051169	wsnp_Ex_c24933_34187952	3.1
QSzWd.crc-5B.1	SzWdB04A	5B	59.4	57.7–59.8	10.84	7.1	0.7336	wsnp_Ku_c17875_27051169	wsnp_Ex_c24933_34187952	3.1
QArPe.crc-5B.2	ArPeG00A	5B	130.3	127.0–132.5	3.56	2.7	0.0917	tPt-3144	wsnp_BE446509B_Ta_2_6	3.1
QDMin.crc-5B.2	DMinG00A	5B	131.7	127.0–132.5	3.08	2.0	0.2918	tPt-3144	wsnp_BE446509B_Ta_2_6	3.1
QSzWd.crc-5B.2	SzWdG00A	5B	131.9	127.0–132.5	3.50	2.6	0.3167	tPt-3144	wsnp_BE446509B_Ta_2_6	3.0
QAMiL.crc-5B.2	AMiLB04A	5B	139.7	139.0–144.0	3.80	2.7	0.4339	Excalibur_c92555_283	tplb0051n17_791	3.0
QDMin.crc-5B.2	DMinB04A	5B	139.7	139.0–144.0	4.29	2.6	0.4458	Excalibur_c92555_283	tplb0051n17_791	3.1
QSzWd.crc-5B.2	SzWdB04A	5B	139.7	139.0–144.0	5.53	3.4	0.5057	Excalibur_c92555_283	tplb0051n17_791	3.1
QRect(var).crc-5B	RectB04S	5B	157.0	154.0–162.3	3.41	3.9	-0.0015	wsnp_JD_c12221_12509984	RAC875_c17841_242	3.2
QRect(var).crc-5B	Rect2YRS	5B	164.1	162.2–164.1	3.55	3.6	-0.0013	Kukri_c3070_72	Tdurum_contig43552_666	3.0
QAMaL.crc-5D	AMaLB04A	5D.2	55.8	52.3–65.6	7.06	8.5	-1.218	gdm63	BS00088592_51	3.1
QPer.crc-5D	PerB04A	5D.2	56.1	47.2–71.3	3.77	4.0	-1.9431	gdm63	BS00088592_51	3.1
QAsym.crc-5D	AsymB04A	5D.2	58.7	52.3–72.7	4.35	6.8	-1.8617	gdm63	BS00088592_51	3.0
QAsym.crc-5D	Asym2YRA	5D.2	61.6	49.0–77.4	3.95	4.6	-1.5365	gdm63	BS00088592_51	3.0
QDMax.crc-5D	DMax2YRA	5D.2	63.5	50.9–77.5	4.34	5.6	-0.9869	gdm63	BS00088592_51	3.2
QAMaL.crc-5D	AMaL2YRA	5D.2	81.7	81.0–82.6	3.80	3.6	-0.761	wsnp_Ku_c46270_53051831	Excalibur_c20024_806	3.1
QArea(var).crc-5D	AreaB04S	5D.2	88.8	87.5–88.8	4.46	6.5	-14.9761	wsnp_Ex_c5185_9189184	Lr1	3.0
QDMax.crc-5D	DMaxB04A	5D.2	88.8	87.5–88.8	3.25	4.3	-0.8925	wsnp_Ex_c5185_9189184	Lr1	3.1
QSzLn.crc-5D	SzLnB04A	5D.2	88.8	87.5–88.8	3.23	4.3	-0.8955	wsnp_Ex_c5185_9189184	Lr1	3.0
QDMen.crc-6B	DMen2YRA	6B	138.3	134.4–142.1	3.52	3.4	0.4556	Kukri_c16404_100	RAC875_c6813_168	3.1
QArea.crc-6B	Area2YRA	6B	138.4	134.4–142.1	3.71	3.8	51.0205	RAC875_c6813_168	BS00049082_51	3.0
QArPe.crc-6B	ArPe2YRA	6B	139.0	135.4–142.1	3.97	2.7	0.0954	RAC875_c6813_168	BS00049082_51	3.1
QArPe.crc-6B	ArPeB04A	6B	139.0	135.4–142.1	4.41	3.5	0.1299	RAC875_c6813_168	BS00049082_51	3.0
QDMen.crc-6B	DMenB04A	6B	139.2	135.6–142.1	5.30	4.5	0.608	RAC875_c6813_168	BS00049082_51	3.0
QArea.crc-6B	AreaB04A	6B	139.4	134.5–142.1	4.51	4.6	64.4603	RAC875_c6813_168	BS00049082_51	3.0
QPer.crc-6B	PerB04A	6B	139.6	133.3–142.1	4.78	4.9	2.1552	RAC875_c6813_168	BS00049082_51	3.1
QAsym(var).crc-6D	AsymB04S	6D.1	53.7	52.0–55.1	4.34	2.3	-0.4217	Ku_c13130_1319	BS00047195_51	3.1
QDMax.crc-6D	DMaxB04A	6D.1	53.8	52.0–55.1	4.04	5.4	-1.0019	Ku_c13130_1319	BS00047195_51	3.1
QSzLn.crc-6D	SzLnB04A	6D.1	53.8	52.0–55.1	4.05	5.4	-1.0091	Ku_c13130_1319	BS00047195_51	3.0
QAMaL.crc-6D	AMaL2YRA	6D.1	53.9	52.0–55.1	9.02	9.2	-1.2132	BS00047195_51	D_GDRF1KQ02FFPXT_243	3.1
QAMiL.crc-6D	AMiLG00A	6D.1	64.8	64.2–65.5	6.95	4.8	-0.4392	RAC875_c18002_58	wsnp_Ex_c37749_45436366	3.1
QArPe.crc-6D	ArPe2YRA	6D.1	64.8	64.2–66.1	3.63	2.3	-0.0888	RAC875_c18002_58	wsnp_Ex_c37749_45436366	3.1
QArPe.crc-6D	ArPeG00A	6D.1	64.8	64.2–65.4	6.84	5.1	-0.1268	RAC875_c18002_58	wsnp_Ex_c37749_45436366	3.1
QSzWd.crc-6D	SzWd2YRA	6D.1	64.8	64.2–66.3	3.47	2.5	-0.3291	RAC875_c18002_58	wsnp_Ex_c37749_45436366	3.1
QSzWd.crc-6D	SzWdG00A	6D.1	64.8	64.2–65.5	6.94	5.3	-0.4547	RAC875_c18002_58	wsnp_Ex_c37749_45436366	3.0
QArea.crc-6D	AreaG00A	6D.1	64.9	64.8–66.2	8.94	9.5	-74.2415	wsnp_Ex_c37749_45436366	barc273	3.1
QDMen.crc-6D	DMenG00A	6D.1	64.9	64.8–65.9	7.29	6.2	-0.5715	wsnp_Ex_c37749_45436366	barc273	3.0
QDMin.crc-6D	DMinG00A	6D.1	64.9	64.8–65.7	6.98	4.4	-0.4352	wsnp_Ex_c37749_45436366	barc273	3.1
QArea.crc-6D	Area2YRA	6D.1	65.7	64.8–68.6	4.76	5.2	-59.5886	wsnp_Ex_c37749_45436366	barc273	3.0
QDMen.crc-6D	DMen2YRA	6D.1	65.7	64.8–68.9	4.46	4.7	-0.5298	wsnp_Ex_c37749_45436366	barc273	3.1
QAsym.crc-6D	AsymG00A	6D.1	65.8	64.8–66.3	7.84	9.1	-2.1758	wsnp_Ex_c37749_45436366	barc273	3.1
QDMin.crc-6D	DMin2YRA	6D.1	65.8	64.8–68.9	3.62	3.1	-0.39	wsnp_Ex_c37749_45436366	barc273	2.9
QAMiL(var).crc-6D	AMiL2YRS	6D.1	66.0	64.8–68.9	3.53	5.4	-0.072	wsnp_Ex_c37749_45436366	barc273	3.2
QArea.crc-6D	AreaB04A	6D.1	66.0	64.8–68.9	3.05	3.0	-52.3209	wsnp_Ex_c37749_45436366	barc273	3.0
QDMin(var).crc-6D	DMin2YRS	6D.1	66.0	64.8–68.9	3.10	5.6	-0.0727	wsnp_Ex_c37749_45436366	barc273	3.0
QPer.crc-6D	Per2YRA	6D.1	66.1	64.8–68.9	3.54	4.5	-1.9293	wsnp_Ex_c37749_45436366	barc273	3.0
QSzWd(var).crc-6D	SzWd2YRS	6D.1	66.2	64.9–68.9	3.42	7.2	-0.0752	wsnp_Ex_c37749_45436366	barc273	3.1
QPer.crc-6D	PerB04A	6D.1	66.3	65.1–68.4	5.14	5.1	-2.1925	wsnp_Ex_c37749_45436366	barc273	3.1
QAMaL.crc-6D	AMaLB04A	6D.1	66.4	65.1–68.7	4.86	5.1	-0.9457	barc273	BS00021881_51	3.1
QArea(var).crc-6D	Area2YRS	6D.1	66.4	65.3–68.5	3.63	6.0	-11.394	barc273	BS00021881_51	3.0
QAsym.crc-6D	Asym2YRA	6D.1	66.4	65.3–68.4	8.13	8.5	-2.0773	barc273	BS00021881_51	3.0
QAsym.crc-6D	AsymB04A	6D.1	66.4	65.3–68.7	5.66	7.7	-1.9888	barc273	BS00021881_51	3.0
QAsym(var).crc-6D	Asym2YRS	6D.1	66.4	65.0–68.8	3.32	4.6	-0.3368	barc273	BS00021881_51	3.1
QDMax.crc-6D	DMax2YRA	6D.1	66.4	65.1–68.4	6.99	7.6	-1.147	barc273	BS00021881_51	3.2
QDMax.crc-6D	DMaxG00A	6D.1	66.4	65.4–68.5	7.42	9.3	-1.2619	barc273	BS00021881_51	3.1
QAMaL.crc-6D	AMaLG00A	6D.1	66.5	65.3–68.6	7.52	8.7	-1.1501	barc273	BS00021881_51	3.1
QPer.crc-6D	PerG00A	6D.1	66.6	65.0–68.9	5.97	6.9	-2.3155	barc273	BS00021881_51	3.1
QSzLn.crc-6D	SzLn2YRA	6D.1	66.7	65.3–68.9	5.21	5.7	-0.9848	barc273	BS00021881_51	3.1
QSzLn.crc-6D	SzLnG00A	6D.1	66.9	65.1–68.9	4.59	6.1	-1.0146	barc273	BS00021881_51	3.1
QDMax.crc-7A	DMaxB04A	7A	139.4	138.3–140.5	3.37	4.4	-0.916	Excalibur_c8066_791	wsnp_Ex_c9476_15710162	3.1
QPer.crc-7A	Per2YRA	7A	139.4	138.3–140.5	3.14	3.8	-1.7931	Excalibur_c8066_791	wsnp_Ex_c9476_15710162	3.0
QPer.crc-7A	PerG00A	7A	139.4	138.3–140.5	4.13	4.6	-1.9008	Excalibur_c8066_791	wsnp_Ex_c9476_15710162	3.1
QSzLn.crc-7A	SzLn2YRA	7A	139.4	138.3–140.5	4.00	4.2	-0.8525	Excalibur_c8066_791	wsnp_Ex_c9476_15710162	3.1
QSzLn.crc-7A	SzLnG00A	7A	139.4	138.3–140.5	4.82	6.2	-1.0314	Excalibur_c8066_791	wsnp_Ex_c9476_15710162	3.1
QSzLn.crc-7A	SzLnB04A	7A	139.4	138.3–140.5	3.41	4.5	-0.9274	Excalibur_c8066_791	wsnp_Ex_c9476_15710162	3.0
QAMaL.crc-7A	AMaLB04A	7A	154.7	153.5–156.8	4.46	4.7	-0.9134	wmc809	BS00009886_51	3.1
QAsym.crc-7A	Asym2YRA	7A	154.7	153.5–156.8	5.12	5.1	-1.6306	wmc809	BS00009886_51	3.0
QAsym.crc-7A	AsymB04A	7A	154.7	153.5–156.8	3.11	4.1	-1.4637	wmc809	BS00009886_51	3.0
QDMax.crc-7A	DMax2YRA	7A	154.7	153.6–156.8	6.93	7.5	-1.1542	wmc809	BS00009886_51	3.2
QPer.crc-7A	PerB04A	7A	154.7	153.5–156.8	3.41	3.2	-1.7728	wmc809	BS00009886_51	3.1
QAMaL.crc-7A	AMaL2YRA	7A	154.8	153.8–156.8	7.85	7.9	-1.1332	BS00009886_51	BS00068055_51	3.1
QArea.crc-7A	AreaG00A	7A	155.0	153.5–156.8	3.21	3.2	-43.8849	BS00009886_51	BS00068055_51	3.1
QDMax.crc-7A	DMaxG00A	7A	155.4	153.7–156.8	5.36	6.5	-1.073	BS00009886_51	BS00068055_51	3.1
QAMaL.crc-7A	AMaLG00A	7A	156.4	153.7–156.8	6.04	6.8	-1.0352	BS00009886_51	BS00068055_51	3.1
QAsym.crc-7A	AsymG00A	7A	157.3	156.7–157.3	3.52	3.8	-1.4281	BS00068055_51	Excalibur_c3476_691	3.1
QSphr.crc-7A	SphrG00A	7A	157.3	156.7–157.3	3.09	4.3	0.0036	BS00068055_51	Excalibur_c3476_691	3.1

^a^ Trait, environment (G00 = Glenlea 2000, B04 = Brandon 2004), statistic (A = average, S = standard deviation, var = variance/variability). e.g. AMaLG00A is the average Major Axis Length in Glenlea 2000.

^b^ Confidence interval determined by one LOD drop-off.

^c^ Additive effect of allele substitution. The units are those of the respective trait. A positive sign indicated that the ‘AC Domain’ allele increased the respective quantitative trait, and vice-versa.

ICIM-epistasis (QICE module) identified a small number of epistatic QTL, which are reported in S4 Table. For seed shape traits, epistatic QTL were detected for Area, ArPe, DMen, DMin, Rect, SzWd, variability of Area, variability of AmiL, and variability of DMen. Epistatic QTL were also detected for Plht, Gwt, Twt, and Fyd. The epistatic interaction between chromosome 1D at 38 cM and chromosome 6B at 84 cM was detected more consistently than the others. This epistatic interaction involved Gwt, Area, ArPe, and DMen, which intuitively should be correlated. Interestingly, additive effect QTL were not detected on chromosome 1D at 38 cM or chromosome 6B at 84 cM using the QIC module for any trait. The remaining epistatic interactions were not consistently identified in different seed shape traits and/or between different datasets (i.e. environments).

### Chromosome 1A

QTL for ArPe (*QArPe*.*crc-1A*), DMen (*QDMen*.*crc-1A*), DMin (*QDMin*.*crc-1A*), Per (*QPer*.*crc-1A*), SzLn (*QSzLn*.*crc-1A*), and SzWd (*QSzWd*.*crc-1A*) had LOD peaks from 0 to 23.5 cM. These QTL had relatively low LOD scores and were mainly detected in the Glenlea 2000 dataset. The ‘AC Domain’ allele decreased each of these seed shape traits. No other QTL for other traits (Gwt, Twt, Fyd) were identified on this chromosome.

### Chromosome 1B

A flour yield QTL *QFyd*.*crc-1B* mapped to linkage group 1B.1 with LOD peaks at 81.7 and 92.1 cM, while three marginally significant seed shape QTL had LOD peaks nearby (*QDMen*.*crc-1B*, *QArea(var)*.*crc-1B*, *QArPe(var)*.*crc-1B*). The ‘AC Domain’ allele decreased Fyd in the region.

### Chromosome 1D

On chromosome 1D, the most statistically significant QTL peaks for grain shape traits (*QAMaL*.*crc-1D*, *QAsym*.*crc-1D*, *QDMax*.*crc-1D*, *QPer*.*crc-1D*, *QSzLn*.*crc-1D*, *QAsym(var)*.*crc-1D*) were located within a 23 cM interval (79.3–102.3 cM). These QTL were located near a Twt QTL with LOD peaks located between 96.9 and 117.3 cM. No Gwt QTL was identified on chromosome 1D. Based on the additive effects of these QTL (Tables 2 and 3), the ‘AC Domain’ allele increased kernel length, Asym, and Per, and decreased Twt. The changes in kernel shape as a result of variation in this region of chromosome 1D appear to affect packing efficiency of kernels (i.e. test weight).

### Chromosome 2A

QTL analysis identified a Twt QTL on chromosome 2A with LOD peaks at 89.6 cM with the ‘AC Domain’ allele decreasing Twt. No other QTL were identified in this genomic region.

### Chromosome 2B

Three QTL clusters were present on chromosome 2B (linkage group 2B.1). QTL for grain width (*QAMiL*.*crc-2B*.*1*, *QDMin*.*crc-2B*.*1*, *QSzWd*.*crc-2B*.*1*) and ArPe (*QArPe*.*crc-2B*.*1*) were approximately located at 37 cM, which is the location of *QTwt*.*crc-2B* at 33 cM. The ‘AC Domain’ allele reduced Twt and grain width (Tables 2 and 3). This same region resulted in variability in Rect and Rndn. In addition, grain roundness QTL (*QRect*.*crc-2B*, *QRndn*.*crc-2B*, *QSphr*.*crc-2B*), kernel width (*QAMiL*.*crc-2B*.*2*, *QDMin*.*crc-2B*.*2*, *QSzWd*.*crc-2B*.*2*), and ArPe (*QArPe*.*crc-2B*.*2*) were located at 66 cM, which was also the location of *QGwt*.*crc-2B*.*1*. The ‘AC Domain’ allele decreased Gwt, Rect, Sphr, AMiL, DMin, SzWd, and ArPe, but increased Rndn. Finally, QTL for grain size (*QArea*.*crc-2B*) and mean kernel diameter (*QDMen*.*crc-2B*.*3*) was located at approximately 91 cM, which was near the location of another grain weight QTL *QGwt*.*crc-2B*.*2* at 87 cM. The ‘AC Domain allele’ decreased Gwt, grain area, and mean kernel diameter.

### Chromosome 2D

QTL for grain shape traits Rect, Rndn, and Sphr (*QRect*.*crc-2D*, *QRndnd*.*crc-2D*, and *QSphr*.*crc-2D*) were identified at approximately 87 cM. QTL for variation in kernel width mapped nearby at 102 cM. A *Twt* QTL (*QTwt*.*crc-2D*) was also identified at 88.9 cM (Brandon 2004 dataset) and at 102.7 cM (Morden 1999 dataset).

### Chromosome 3A

A strong QTL for variability in Sphericity (Sphr) was consistently detected at 65.9 cM. The ‘AC Domain’ allele increased variability in this trait. No other QTL were detected on chromosome 3A.

### Chromosome 3B

Two QTL regions were detected on chromosome 3B. The first region, located at 0.0–0.3 cM, affected Gwt and seed shape traits (Area, Per, ArPe, AMiL, DMen, DMin, and SzWd). The ‘AC Domain’ allele decreased Gwt and the seed shape traits. In the second QTL region on chromosome 3B, QTL for grain shape (*QAsym*.*crc-3B*), *Twt* (*QTwt*.*crc-3B*), and *Fyd* (*QFyd*.*crc-3B*) were identified within a 14.4 cM region (60.1–74.5 cM). This genetic interval also contained a KASP marker *Kasp3B (survey)_17*, which was developed from a survey sequence SNP associated with pre-harvest sprouting resistance (PHS) on chromosome 3B [39]. *QTwt*.*crc-3B* has been previously reported [27], and was flanked by SSR markers *wmc625* and *barc164*, located at 61.9 and 69.0 cM, respectively [39]. Similarly, *QFyd*.*crc-3B* mapped to *wmc446* [46] and is located at position 61.9 cM on chromosome 3B [39]. A positive additive effect for Asym on 3B could be interpreted as the ‘AC Domain’ allele contributing to grain shape asymmetry (Asym), which in turn might have resulted in the reduction in Twt and increased Fyd associated with this region. QTL for variability in Area, Per, ArPe, Asym, AMiL, DMen, DMin, SzWd were detected in this region with the ‘AC Domain’ increasing variability in these traits.

### Chromosome 3D

On linkage group 3D.2, QTL for grain shape (numerous QTL), Twt (*QTwt*.*crc-3D*), Gwt (*QGwt*.*crc-3D*), and Fyd (*QFyd*.*crc-3D*) were detected in two main regions. Twt, Fyd, AmaL, and DMax QTL had LOD peaks within a 5.1 cM interval (77.1–82.8 cM), in which the ‘AC Domain’ allele decreased each of these traits. The Gwt QTL mapped to a different location with QTL peaks ranging 4.9–20.9 cM. QTL for Area, Per, ArPe, AMiL, DMen, DMin, SzLn, and SzWd also mapped to this same general region with LOD peaks ranging between 2.3 and 35.5 cM. Again, the ‘AC Domain’ allele decreased each of these traits.

### Chromosome 4A

Numerous QTL were detected on chromosome 4A. A Gwt QTL was detected at 90.1 cM along with QTL for AMaL, AMiL, Area, ArPe, Asym, DMax, DMen, DMin, Per, SzLn, and SzWd, and variability in AMaL, Area, Asym, DMen, and Per. The ‘AC Domain’ allele increased each of these traits. Grain shape QTL were detected at 38 cM for the traits AMaL, Asym, DMax, Per, Rect, Rndn, Sphr, and SzLn. The ‘AC Domain allele’ increase AMaL, Asym, DMax, Per, Rndn, and SzLn, but decreased Rect and Sphr. At 63 cM, QTL for AMiL and DMin, and variation in Area, ArPe, and DMen were detected in the Glenlea 2000 dataset. The QTL located at 38 and 63 cM had no detectable effect on Gwt, Twt, or Fyd.

### Chromosome 4B

**Fig 1:**
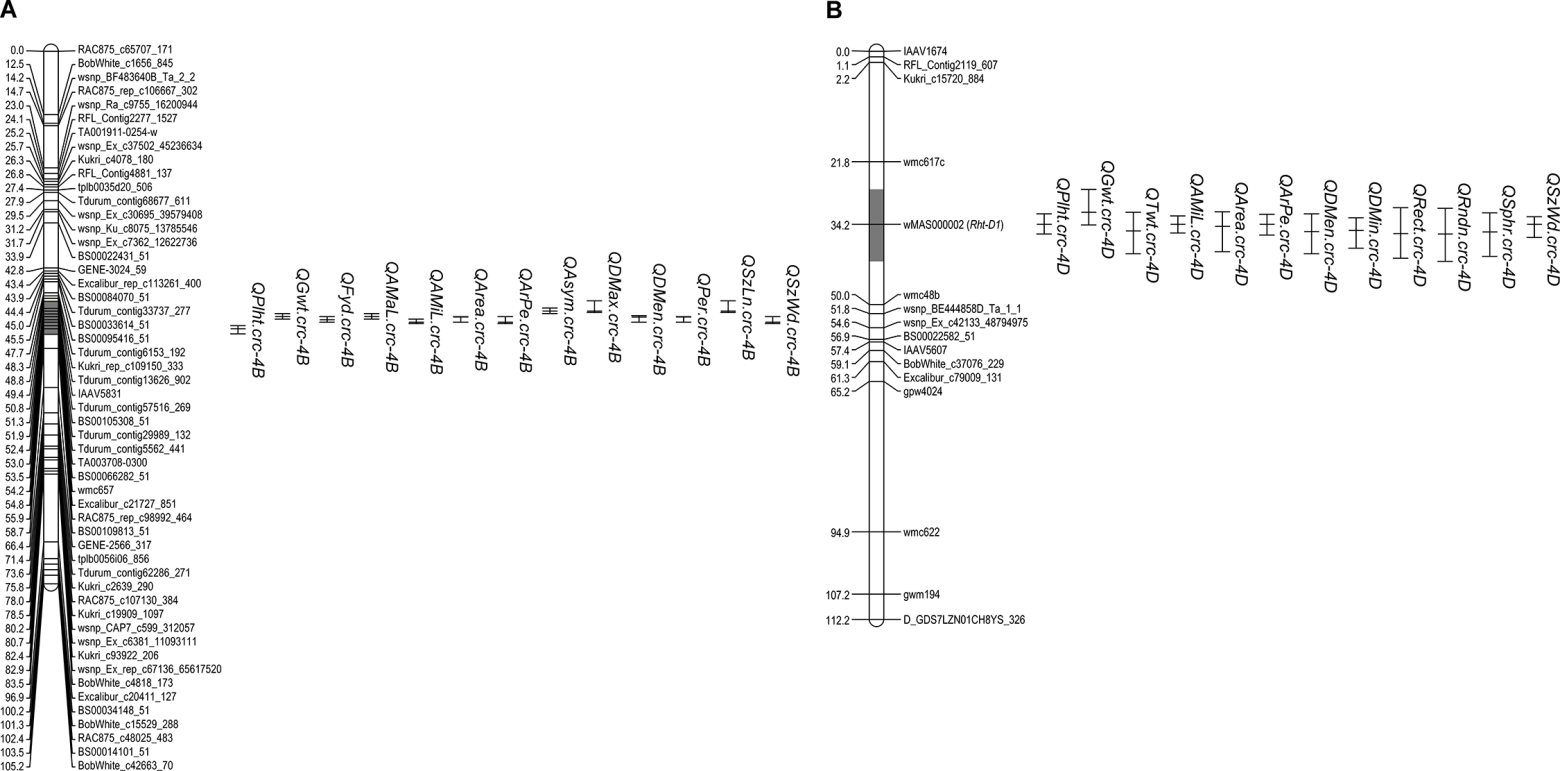
Plht, Gwt, Twt, Fyd, and the most significant seed shape QTL (LOD peaks > 10), and their 1 LOD drop-off confidence intervals, identified in the RL4452/‘AC Domain’ DH population on (A) chromosome 4B within a 6.5 cM region (in gray) and (B) chromosome 4D within a 14.2 cM region (in gray).

On chromosome 4B, a 4.5 cM interval (51.4–55.9 cM) coincided with the LOD peak locations of a major QTL for Plht (*QPlht*.*crc-4B*), Gwt (*QGwt*.*crc-4B*), and Fyd (*QFyd*.*crc-4B*) (Fig 1). In addition, a significant QTL was detected in this region of chromosome 4B for each of the 14 seed shape traits, and for variability in AMaL, AMiL, Area, ArPe, Asym, DMax, DMen, DMin, Per, SzLn, and SzWd. When considering 1 LOD drop-off confidence intervals for these QTL, this narrow genetic region corresponds to a very large ~601 Mbp physical region in the IWGSC Chinese Spring RefSeq v1.0 (S1 Table). The interval includes the centromere and appears to be an area of low recombination. The HighConfidenceGenesv1.0 track in JBrowse indicates that 2,979 high confidence genes are present in this interval. The diagnostic SNP marker *wMAS000001* for the *Rht-B1* locus (http://www.cerealsdb.uk.net/cerealgenomics/CerealsDB/kasp_download.php?) was tested on the RL4452/‘AC Domain’ population to determine if *Rht-B1* was responsible for this QTL region, but the marker was monomorphic in the population. In the IWGSC Chinese Spring RefSeq v1.0 sequence, *Rht-B1* is physically located between 30,861,382 to 30,863,247 bp (around 40 cM on the 4B linkage map in this cross). The ‘AC Domain’ allele reduced Plht, Gwt, Fyd, grain length, grain width, grain area, etc. (Tables 2 and 3).

### Chromosome 4D

On chromosome 4D, a 4.9 cM genomic region (31.8–36.7 cM) coincided with the LOD peaks of QTL for Plht (*QPlht*.*crc-4D*), Gwt (*QGwt*.*crc-4D*), and Twt (*QTwt*.*crc-4D*). Also in the same region with QTL peaks ranging from 34.2–39.9 cM were QTL for AMiL, Area, ArPe, DMen, DMin, Per, Rect, Rndn, Sphr, and SzWd, and QTL for variability in Rect and Rndn. The diagnostic SNP marker *wMAS000002* for the *Rht-D1* locus mapped to 34.2 cM, which is the predicted location for all of these QTL. The ‘AC Domain’ allele increased *Plht*, *Gwt*, *Twt*, grain length, grain width, grain area, Rect, and Sphr, while decreased Rndn and variability in Rect and Rndn (Tables 2 and 3). RL4452 (‘Glenlea’*6/‘Kitt’) carries the *Rht-D1b* allele from the Minnesota spring wheat variety ‘Kitt’. Nearby a QTL for Rndn (*QRndn*.*crc-4D*.*2*), Sphr (*QSphr*.*crc-4D*.*2*), Rect (*QRect*.*crc-4D*.*2*), and variability in Asym (*QAsym(var)*.*crc-4D*) was detected at 53 cM.

### Chromosome 5B

QTL for grain width, Area, Per, and ArPe were detected at approximately 59 cM on chromosome 5B. In addition, a second QTL for grain width was detected at 131 cM based upon the Glenlea 2000 dataset, and at 140 cM based upon the Morden 2004 dataset. A QTL for variability in Rect was identified at 161 cM. The ‘AC Domain’ alleles increased grain width in these two QTL regions. Interestingly, there were no QTL for Gwt, Twt, or Fyd detected on chromosome 5B.

### Chromosome 5D

A cluster of QTL affecting AMaL (*QAMaL*.*crc-5D*), Asym (*QAsym*.*crc-5D*), DMax (*QDMax*.*crc-5D*), Per (*QPer*.*crc-5D*), SzLn (*QSzLn*.*crc-5D*), and variability in Area (*QArea(var)*.*crc-5D*) were identified on linkage group 5D.2 with LOD peaks from 55.8 to 88.8 cM. The ‘AC Domain’ allele decreased each of these traits. No other QTL were detected on chromosome 5D.

### Chromosome 6B

A Gwt QTL (*QGwt*.*crc-6B*) was detected on chromosome 6B with LOD peaks located at 139.4 and 159.1 cM. QTL for Area (*QArea*.*crc-6B*), ArPe (*QArPe*.*crc-6B*), DMen (*QDMen*.*crc-6B*), and Per (*QPer*.*crc-6B*) were also identified at 139 cM. In this region, the ‘AC Domain allele’ increased Gwt and the four seed shape traits.

### Chromosome 6D

QTL for a number of grain shape traits had LOD peaks mainly located within a 2.1 cM interval (64.8–66.9 cM) on linkage group 6D.1. However in four instances, the LOD peaks for the same traits were located at 54 cM. The ‘AC Domain’ allele decreased grain length (AMaL, DMax, SzLn), grain width (AMiL, DMin, SzWd), Per, Asym, and Area. No QTL for Gwt, Twt, or Fyd were detected in this region.

### Chromosome 7A

QTL peaks for grain shape (*QAMaL*.*crc-7A*, *QArea*.*crc-7A*, *QAsym*.*crc-7A*, *QDMax*.*crc-7A*, *QPer*.*crc-7A*, *QSphr*.*crc-7A*, *QSzLn*.*crc-7A*) were identified in a 17.9 cM interval (139.4–157.3 cM), while a Twt QTL (*QTwt*.*crc-7A*) was located at 84.1 cM. Since these QTL regions were not closely linked, it is assumed that one QTL predominantly affects Twt and the other seed shape (i.e. at least two genes control this variability). For *QTwt*.*crc-7A*, the ‘AC Domain’ allele increased test weight. For the grain shape QTL region, the ‘AC Domain’ allele reduced grain length, Area, Asym, Per, and Sphr. No Gwt or Fyd QTL were identified on 7A.

### Chromosome 7D

The major flour yield QTL *QFyd*.*crc-7D* was not coincident with QTL for Gwt, Twt, or seed shape, but was coincident with a major maturity QTL *QMat*.*crc-7D* previously identified in this population [27]. *QFyd*.*crc-7D* was a broad QTL with the main peak located at 16.2 cM and secondary peaks located at 31.9 and 43.1 cM based on interval mapping (IM-ADD, S1 Fig). Based on these data, it is possible that *QFyd*.*crc-7D* is the result of two or more linked genes. Interestingly, the maturity QTL *QMat*.*crc-7D* has a single peak at 19.6 cM and no secondary peaks (S1 Fig). No seed shape QTL were identified on chromosome 7D.

## Discussion

The objectives of our study were to identify significant grain shape and agronomic trait QTL, and determine their interrelationships. SNPs from a wheat 90K Infinium Custom Beadchip were previously used to generate a high density linkage map of a RL4452/‘AC Domain’ DH population [39], which in turn was used to identify QTL for the above traits. QTL were identified on chromosomes 1A, 1B, 1D, 2B, 2D, 3A, 3B, 3D, 4A, 4B, 4D, 5B, 5D, 6B, 6D, 7A (grain shape); 4B, 4D (Plht); 2B, 3B, 3D, 4A, 4B, 4D, 6B (Gwt); 1D, 2A, 2B, 2D, 3B, 3D, 4D, 7A (Twt); and 1B, 3B, 3D, 4B, 7D (Fyd). The most significant QTL for grain shape, Plht, and Gwt were detected on chromosomes 4B and 4D. The most significant Twt QTL were identified on 3B and 4D. The most significant Fyd was located on chromosome 7D, but another important Fyd QTL coincided with the Twt QTL on chromosome 3B.

A major QTL for grain shape, Gwt, Fyd, and Plht mapped to a narrow genetic region on chromosome 4B, which corresponds to the centromere and a very large portion of the chromosome. ‘AC Domain’ alleles contributed to a reduction in Plht, Gwt, Fyd, and grain size, in addition to negative additive effect values for grain shape traits. The same Gwt QTL was identified on 4B, and is associated with SSR marker *wmc238* [27], located at 51.9 cM. In our study, *wmc238* was located 0.5 cM from *Tdurum_contig5562_441*, positioned at 52.4 cM on chromosome 4B [39]. Further, the Plht QTL of both studies mapped essentially to the same position based upon the linked SSR marker *gwm513* that co-segregates with *TA003708-0300*. Markers *gwm513* and *TA003708-0300* were 0.6 cM apart from the grain shape and Gwt QTL peak SNP marker *Tdurum_contig5562_441* of our study. Therefore, all SNP and SSR markers within this narrow region on 4B may be useful for MAS of grain size, Gwt, and Plht traits in germplasm and breeding material. *QPlht*.*crc-4B*, *QGwt*.*crc-4B*, *QFyd*.*crc-4B*, and the many seed shape QTL in this region overlap with the ‘QTL region 15’ in the cross ND705/PI 414566, which affects Twt, Gwt, kernel area, and kernel length [10]. The grain shape, Fyd, and Gwt QTL in the RL4452/‘AC Domain’ are likely the result of pleiotrophy of the reduced plant height ‘AC Domain’ allele at *QPlht*.*crc-4B*.

The plant height QTL *QPlht*.*crc-4B* was previously believed to be the result of segregation at the *Rht-B1* locus [27]. However, the improved SNP-based linkage map revealed that *QPlht*.*crc-4B* mapped proximal of the expected location of *Rht-B1* (possibly on 4BL), which could not be resolved based on the older SSR map [27]. It is important to note that the RL4452/‘AC Domain’ mapping population was also monomorphic for the KASP marker *wMAS000001*, a diagnostic marker for *Rht-B1*. *Rht-B1* is also physically located outside the confidence interval for these QTL. *QPlht*.*crc-4B* and the other linked/pleiotropic QTL must not be the result of segregation at the *Rht-B1* locus. Based upon the BLAST locations of the SNP markers in the 4B linkage map and 1 LOD drop-off confidence intervals for these QTL, this region contains 2,979 high confidence genes in the IWGSC Chinese Spring RefSeq v1.0. Additional research is needed to identify candidate genes responsible for these QTL.

The genetic interval on chromosome 4D flanked by SSR markers *wmc617* and *wmc48* was found to carry the most significant QTL for seed shape, Plht, Gwt, and Twt. *Rht-D1* mapped to this centre of this region as indicated by the diagnostic SNP marker *wMAS000002*. RL4452 carries the dwarfing allele *Rht-D1b*, which reduced Plht, Gwt, Twt, grain width, and Area, but had no detectable effect on Fyd. *Rht-D1b* also negatively impacted the grain shape traits Rect and Sphr, and the net result of these changes in kernel shape was a reduction in Twt. Our findings are in agreement with those previously reported for the same RL4452/‘AC Domain’ population mapped using 369 SSR markers [27, 46]. Based on these results, it is likely that the variation in seed shape near *Rht-D1* is due to its pleiotropic effects.

Chromosome 4A is particularly interesting in this population. Three QTL regions were identified in this study affecting seed size and shape. QTL for grain length (*AMaL*, *DMax*, *SzLn*), *Per*, *Rect*, and *Sphr* mapped to 38 cM on chromosome 4A. These QTL were supported by the identification of the same QTL region (*Twt*, *Gwt*, kernel area) in the cross ND705/PI 414566 (*Twt*, *Gwt*, kernel area) [10] and in Synthetic/Opata (vertical perimeter) [7]. QTL for grain width (*AMiL*, *DMin*) and variability for kernel parameters within samples were detected at 63 cM. Likewise, a QTL for length-width ratio (*QKLWR*.*ndsu*.*4A*.*1*) was detected in the same region [10]. Finally, a QTL for grain weight *QGwt*.*crc-4A* mapped to 90 cM along with numerous grain shape parameters in the RL4452/‘AC Domain’ population. This was also supported by a second length-width ratio QTL (*QKLWR*.*ndsu*.*4A*.*2*) in the ND705/PI 414566 population [10]. Surprisingly, there were no QTL for *Twt* detected on chromosome 4A in the RL4452/‘AC Domain’ population.

Another interesting locus in the RL4452/‘AC Domain’ population is located on chromosome 7D (linkage group 7D.2). The most important *Fyd* QTL *QFyd*.*crc-7D* mapped to a large interval with predicted locations at 14.4, 23.8, and 43.3 cM based on ICIM. Interval mapping showed multiple peaks in each environment (S1 Fig). Also in this region is a major, days to maturity QTL at 19.6 cM [27, 39], which did not have any secondary peaks (S1 Fig). The presence of multiple peaks for *Fyd* suggested that multiple genes controlling the trait could be located in this region. One of the genes affecting *Fyd* could be the maturity QTL itself. It was hypothesized that this maturity QTL was responsible for a falling number QTL in this region since the parental allele contributing the beneficial additive effect varied between growing environments [39]. Presumably the weather conditions affecting pre-harvest sprouting (i.e. rain and high humidity) varied in different growing seasons. In addition, *QFyd*.*crc-7D* overlaps with *QTKW*.*ndsu*.*7D*, *QTW*.*ndsu*.*7D*, *QKW*.*ndsu*.*7D*, and *QKLWR*.*ndsu*.*7D* [10]. Additional research is needed to clarify the genetic control of these traits at this point in the genome.

A number of other QTL detected in the RL4452/‘AC Domain’ population were also in common with grain shape and size QTL detected in the cross ND705/PI 414566 [10]. *QFyd*.*crc-1B* overlaps with QTL region 2 (*QKW*.*ndsu*.*1B*, *QLWR*.*ndsu*.*1B*.*2*) [10]. The QTL *QTwt*.*crc-2D*, *QRndn*.*crc-2D*, and *QSphr*.*crc-2D* likely overlap with the QTL *QTW*.*ndsu*.*2D*.*1* and *QKLWR*.*ndsu*.*2D*.*1*. *QGwt*.*crc-3D* likely overlaps with the thousand kernel weight QTL *QTKW*.*ndsu*.*3D* and the kernel area QTL *QKA*.*ndsu*.*3D*. Grain width QTL (*QDMin*.*crc-5B* and *QSzWd*.*crc-5B*) overlapped with kernel area and length QTL. Grain length (*AMaL*, *DMax*, *SzLn*), *Asym*, and *Per* QTL of our study mapped to approximately 149 cM on the 7A linkage group, which is consistent with QTL for *Gwt*, kernel length, width, and area [10]. Similarities between QTL on chromosomes 4A, 4B, and 7D in these two populations have already been discussed in the preceding paragraphs. These similarities likely result from shared parentage. ‘AC Domain’ has the pedigree ND499/RL4137//ND585. ND499, ND585, and ND705 are all wheat lines developed by North Dakota State University, so ‘AC Domain’ and ND705 are likely to share many of the same alleles. In addition, the kernel shapes of ‘AC Domain’ and ND705 are similar (short plump kernels), while the kernels of RL4452 and PI 414566 are relatively longer.

## Conclusions

This study identified significant QTL for grain morphology, plant height, grain weight, test weight, and flour yield. Previous QTL studies to identify grain shape have utilized SSRs, DArTs, and other PCR-based markers in segregating populations. In deploying a combination of wheat 90K Infinium SNPs and landmark SSRs, we have been successful in enhancing the marker density on the RL4452/‘AC Domain’ linkage map, and in defining QTL relative to this improved genetic map and the Chinese Spring pseudomolecules. The association between Plht, Gwt, Twt, Fyd, and grain shape QTL confirmed past findings. Genetic analysis of kernel image analysis showed promise, and uncovered additional variation for Gwt, Twt, and Fyd. The per plot heritability estimates were higher for the grain shape traits than Gwt and Fyd, and grain shape QTL were identified that were not associated with Gwt, Twt, and Fyd. Our results should also provide a consensus on the location of linked SNPs and landmark SSRs across maps, which in turn might enable validation of these grain shape QTL in other populations. SNP markers associated with the above traits might also be useful for MAS, and in the identification of candidate genes from rice or other monocots.

## Supporting information

S1 Fig Interval mapping LOD scans for flour yield (Fyd) and time to maturity (Mat) on linkage group 7D.2. Mat data was described previously [27].
(TIF)

S1 Table The RL4452/'AC Domain' linkage map constructed with 193 DH lines tested with 12,202 DNA markers (11,283 SNPs and 919 PCR-based markers). (XLSX)

S2 Table Descriptive statistics and heritability estimates of the traits evaluated on the RL4452/'AC Domain' DH population. (XLSX)

S3 Table Correlation analysis of agronomic, milling, and seed shape traits in the RL4452/‘AC Domain’ DH population. (XLSX)

S4 Table Digenic epistatic QTL identified in the RL4452/‘AC Domain’ DH population by Inclusive Composite Interval Mapping (QICE module) for agronomic, milling, and seed shape traits. (XLSX)
