# Supplementary figures and images for: Relationship between QTL for grain shape, grain weight, test weight, milling yield, and plant height in the spring wheat cross RL4452/‘AC Domain’
Source: PLoS One. 2018 Jan 22;13(1):e0190681. doi: 10.1371/journal.pone.0190681 (PMC5777647; doi:10.1371/journal.pone.0190681)

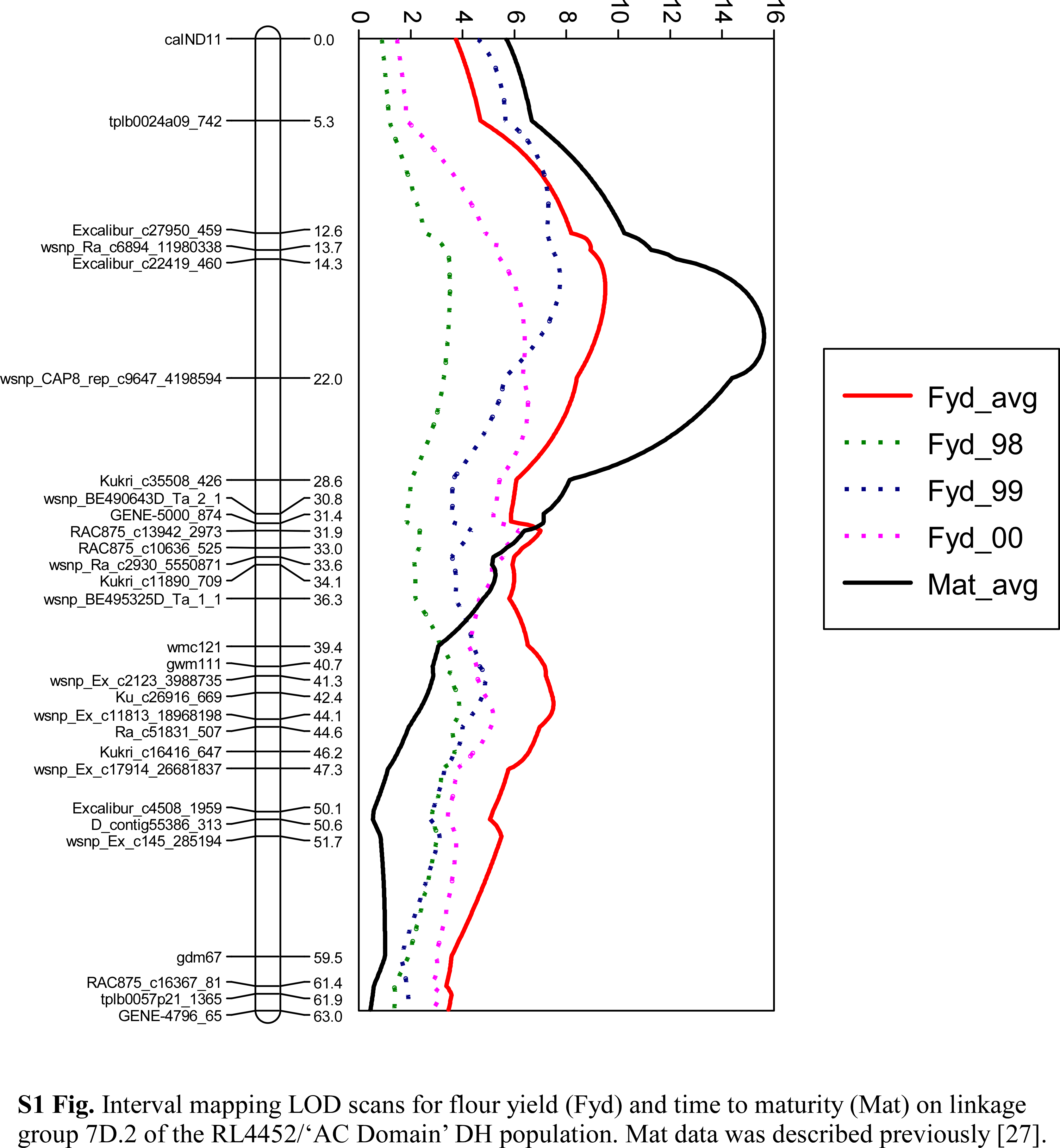

Supplement: S1 Fig — Mat data was described previously [27]. (TIF) [file pone.0190681.s001.tif]
